# Developing a recovery-oriented intervention for people with severe mental illness and an intellectual disability: design-oriented action research

**DOI:** 10.3389/fpsyt.2023.1184798

**Published:** 2023-07-19

**Authors:** Ingeborg Berger, Anne Bruineberg, Margot van Ewijk, Levi de Jong, Michiel van der Hout, Jaap van Weeghel, Lisette van der Meer

**Affiliations:** ^1^Department for Outpatients Severe Mental Health Services, Antes Parnassia Group, Rotterdam, Netherlands; ^2^Creative Media and Game Technology at Hogeschool Rotterdam, Rotterdam, Netherlands; ^3^Department of TRANZO, Tilburg School of Social and Behavioral Sciences, Tilburg University, Tilburg, Netherlands; ^4^Phrenos Center of Expertise on Severe Mental Illness, Utrecht, Netherlands; ^5^Department of Rehabilitation, Lentis Center for Mental Health Care, Zuidlaren, Netherlands; ^6^Department of Clinical and Developmental Neuropsychology, University of Groningen, Groningen, Netherlands

**Keywords:** mild intellectual disability, borderline intellectual functioning, severe mental illness, recovery, strengths

## Abstract

**Introduction:**

Mild intellectual disability or borderline intellectual functioning (MID/BIF) are common in people with severe mental health problems (SMHP). Despite this, there is a lack of treatments adapted for this group of clients.

**Methods:**

This qualitative study describes the development of a new intervention, guided by the principles of action research, for people with SMHP and MID/BIF and mental health professionals to help them talk about all aspects of the process of recovery. The intervention was developed in four cycles and in close cooperation with mental health professionals, experts by experience, other experts in the field of SMHP or MID/BIF, and clients. During all cycles there was a strong focus on the content of the intervention, exercises, understandable language, and drawings for visual support.

**Results:**

This resulted in the intervention “Routes to Recovery,” which covers both complaints and strengths, coping strategies, helpful (social) activities, and how to determine future steps in a recovery plan.

**Discussion:**

Routes to Recovery is a first step in helping professionals and their clients with SMHP and MID/BIF to have a conversation about personal strengths and what the client needs to recover. Future research should investigate the effects of this intervention.

## Introduction

Mild intellectual disability or borderline intellectual functioning (MID/BIF) are common in people with severe mental health problems (SMHP). Research shows that whilst intellectual disability commonly remains unnoticed, it is an important factor in treatment and recovery of people with psychiatric conditions (1–3). There is evidence suggesting that people with intellectual disability may not benefit from recovery oriented care as much as people without intellectual disability (4). Given that there is a paucity in adapted treatments (1), and matched care approaches are often suboptimal (5), this would indicate that there is a need for interventions that are comprehensible for people with MID/BIF in order to improve their chances for recovery.

Current mental health care aims to provide recovery-oriented treatment for all people with SMHP (e.g., psychotic-, bipolar-, and personality disorders, polysubstance abuse, combined with severe dysfunction after a 2 year history of mental health care) (6, 7). Recovery can be defined as a personal process of learning to live better with (severe) mental health problems and it involves more than recovery from the illness itself (8, 9). In recent decades, the recovery movement has become more prominent in Western countries; it states that, rather than just remission of the symptoms of disease, recovery is a journey with many characteristics. It is an active, unique non-linear process with stages or phases, it is a struggle, and most often a life-changing experience (10, 11). Recovery is not only about remission of symptoms of disease, internationally known as clinical recovery, because mental illnesses are often persistent. Therefore it is important for people to learn to live with their vulnerabilities and start a process of strengthening resilience (12). This “personal recovery” as it is termed, is probably the most central and important dimension of the recovery concept (13, 14). The characteristics of the personal recovery process are summarized thoroughly in the CHIME conceptual framework (10). This framework is widely endorsed, and contains the elements of connectedness (e.g., relationships, being part of the community), hope and optimism about the future (e.g., belief in possibility of recovery, motivation to change), identity (e.g., rebuilding/redefining positive sense of identity, overcoming stigma), meaning in life (e.g., meaning of mental illness experiences, spirituality, meaningful life and social roles and goals), and empowerment (personal responsibility, focusing upon strengths, control over life). The optimistic themes of CHIME can be supplemented with the difficult experiences of recovery, CHIME-D, for further understanding and recognizing people’s struggles to recover (15). The longer someone copes with SMHP, the more likely he or she is to forget what it was like to lead a life without illness. For this group it can be difficult to retain self-esteem, and their own values and opinions. It takes time, and courage, to regain, maintain, and appreciate your own strength (16). The interaction between challenges (risks) and resilience factors is critical to ongoing recovery and its maintenance (17).

In recent years, an increasing number of interventions aiming at personal recovery have been introduced, such as Illness Management and Recovery (18), Wellness Recovery and Action Planning (19, 20), “Recovery is up to you” (21), and Toward Recovery and Empowerment and Experiential Expertise (TREE) (22). However, one factor that is important, but often overlooked in treatment and recovery of people with SMHP, including in most of the above-mentioned interventions, is the level of intellectual functioning (4).

MID/BIF, which is characterized by problems with intellectual functioning combined with problems with adaptive functioning, is very common in people with SMHP (23–27). Although extensive international studies are lacking, the prevalence of MID/BIF in people with SMHP is most probably high. Percentages of confirmed or suspected MID/BIF vary from 27% in the outpatient setting for people with common mental health disorders to 42% in community mental health teams clients and admission wards, to almost 67% in long-stay wards. Intellectual deficits caused by cognitive decline are estimated at 7% (1, 28–30). About 34% of the people with intellectual disability have a co-morbid mental disorder. In addition, people with borderline intellectual functioning (BIF) are more likely to suffer from mental disorders and substance misuse (24, 31, 32).

People with MID/BIF experience stress on a daily basis, mostly caused by interpersonal interactions and coping challenges (33). Compared to the general population, people with MID/BIF experience more societal issues, such as difficulty finding a job, and social judgment challenges, making them more socially vulnerable (34). Additionally, they are more likely to be socially isolated, which is a risk factor for mental health problems (35, 36). Nevertheless, they pursue a self-determined life in which they can make their own choices and take responsibilities (37). Professionals face the challenge of looking for appropriate support strategies for persons with MID/BIF and additional mental health problems, with attention and caution about asking too much of clients on the one hand and offering choice and empowerment on the other (38).

In recent years, there has been an increasing interest in adjustments professionals can make in their communication with people with MID/BIF. While understandable communication is crucial for all people, people with MID/BIF in particular would benefit from accessible language solutions (39). Verbal and written language in current mental health treatment is often too difficult for people with SMHP and MID/BIF. Visual support, such as illustrations, pictograms and exercises, is the exception rather than the rule, even though it is a necessity to support understanding and learning in this group (40). Furthermore, non-stigmatizing language is recommended by using person-first rather than disorder-first language for people with SMHP or substance abuse (41). Currently, no literature is available on how to combine non-stigmatizing language with accessible language solutions for people with both SMHP and MID/BIF.

Although people with MID/BIF experience higher rates of mental health problems compared with the general population (42), there is some evidence that people with mild intellectual disability in particular benefit less from regular mental health treatment (4). Reasons could be poor recognition of MID/BIF in clients with SMHP and failure to include a classification or diagnosis in medical files (3). Also, mental health practitioners receive little education in MID/BIF, as a result of which they often feel insufficiently capable of offering treatment to clients with SMHP and MID/BIF, which in turn leads to a reluctance to act (43, 44). Another reason can be the lack of treatment adapted for people with both SMHP and MID/BIF (1). However, there are some inspiring examples. Modifications in group therapy treatment, such as assessing their understanding of difficult concepts (e.g., psychosis, stress, relapse), followed by explanation and improving their knowledge, empowers them and enables them to better identify early signs of illness (45). Furthermore, an intensive personalized rehabilitative support intervention has shown positive results in a treatment for psychosis combined with MID/BIF. Participants improve in multiple areas, such as quality of life, well-being, reductions in unmet needs and increase in global functioning (46). The life story intervention “Who am I?” uses methods from narrative therapy and life review therapy, and was developed for people with both MID/BIF and psychiatric problems. Findings show improvement in experienced mental health problems, in particular for depression, anxiety, obsessive-compulsive complaints and interpersonal sensitivity (47). The intervention “This Is Me” (48) helps people with SMHP to (re)discover their identity and a renewed sense of purpose, and was developed with due consideration for cognitive and communicative disabilities.

Current practice demonstrates that mental health professionals need more detailed guidance to start a conversation about recovery with their clients with both SMHP and MID/BIF. Given the lack of educational and insightful methods that are available for this group of people, the development of training packages regarding the treatment in standard mental health care settings is warranted (1). The current study entails the development of a Dutch recovery-oriented intervention in mental health care for people with both SMHP and MID/BIF. Central questions in the development of this approach include how content, assignments, language use, and visual support should be designed in order to make the intervention understandable, educational, and attractive for people with SMHP and MID/BIF. This would provide guidance to professionals.

## Methods

### Design

The idea of developing the intended intervention was prompted by “a problem” we had experienced. We formulated this problem as follows: in daily professional practice, many clients had a literal and limited understanding of the concept of recovery, namely complete recovery without any remaining complaints (clinical recovery), rather than recovery being the process of understanding one’s personal wishes and strengths despite the remaining symptoms, and being able to lead a meaningful life. Current methods and tools are not sufficient for people with MID/BIF to understand the term “recovery” and be guided through the process. At the same time, professionals lack the tools to guide clients appropriately, which may prevent them from engaging in a conversation about recovery.

We adopted a qualitative method based on the principles of action research to develop an intervention for mental health professionals and clients with SMHP and MID/BIF to discuss the clients’ process of recovery. In action research, the research process itself aims to initiate action, in this case by improving professional practice and at the same time developing knowledge about this improvement by using a cyclical process (plan, act, observe, reflect, revise plan) (49).

We put together a small research team, including a peer support worker, a job coach who also works as an artist, an illustrator with lived experience in mental health, a nurse practitioner and two scientists with expertise in psychiatric rehabilitation and recovery.

The medical ethic commission (METC) Leiden Den Haag Delft gave ethical consent. All participating clients received information about the research in easy, understandable language and provided their informed written consent.

### Setting and population

In Netherlands, people living at home with SMHP often receive treatment from a multidisciplinary team according to the flexible-assertive community treatment (F-ACT) model (50, 51). These “FACT teams” can flexibly vary the intensity of services from assertive outreach (upscaling) to recovery-oriented treatment and rehabilitation (downscaling), according to the clients’ needs. Mental health professionals in a team all share the caseload of that team. Professionals and clients from eight FACT teams participated in the current study. These teams operate in the southern part of Rotterdam, and provide care to about 2000 clients. In the area covered by the participating FACT teams, 63% of the inhabitants have a migration background. Nearly 15% of the residents of this area have a long-term low income (52). Rotterdam has the highest proportion of poor residents in Netherlands (53).

### Procedure and participants

#### Preparation of the intervention

The research team developed a first prototype of the intervention as input for the first round of data collection, based upon the four main topics of interest from the literature: content (what to discuss), assignments, understandable language, and -visual support. This first prototype mainly consisted of text, based on literature about recovery, with explanations about the following subjects (content): mental health issues, what recovery is, different types of recovery, different phases of recovery, elements of CHIME-D (connectedness, hope and optimism, identity, meaning in life, empowerment, and difficulties) and effective strategies (approaches that help). We paid attention to the use of short sentences, to the avoidance of difficult words, and imagery. We also added some example assignments, such as questions about picking up social roles again and what to change, meaningful things in life, true or false questions about functioning in daily life (e.g., “I have fixed appointments during the week: true or false,” with space for written comments. The last assignment, at the end of the prototype, consisted of some questions about recovery, for example “Telling your own story contributes to recovery: true or false?” or “Recovery is the same as healing or getting better: true or false?”) The research team also made some suggestions for drawings, such as showing that a recovery process is not a straight line upwards, the different phases of recovery, and discovering your talents and own strengths.

**Figure 1:**
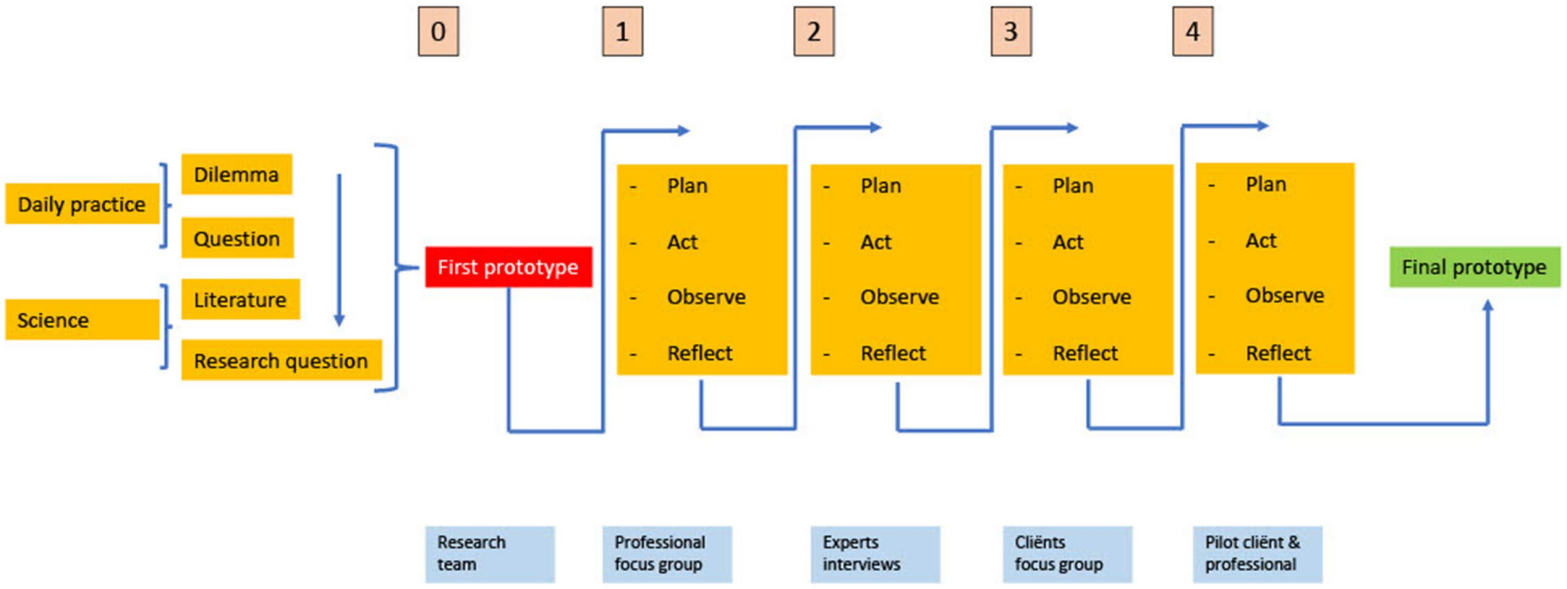
Visualization of design-focused action research [figure based on (54)].

After this preparation, four cycles followed with data collection and interim adjustment of the intervention by the research team. In each of the four cycles, the wheel of plan > action > observe > reflect > plan (49) was followed (Figure 1). A total of 68 participants (professionals *n* = 23, experts *n* = 19, and clients *n* = 26) participated in the course of the study through to the development of the final intervention (Supplementary material 1). The study was conducted in Dutch and English translations are presented in this article for ease of reference.

#### Cycle 1

In this cycle, our research team presented a first prototype of the intervention. We invited eight mental health professionals (Supplementary material 1) from the FACT teams in our organization to join and discuss this first prototype in a focus group. In this first cycle, we aimed to generate further ideas about content, assignments, use of language, and visual support. We used a topic list (Supplementary material 2) for gathering the information. To receive optimal input, we asked professionals with varying professional expertise, but they all had affinity with working with people with MID/BIF in mental health care (selected sample).

We recorded the discussions for data collection and met with the research team to discuss outcomes and adjustments. Subsequently, the research team gathered to reflect on the new information and make adjustments to our preliminary intervention materials.

#### Cycle 2

Data collected in the first focus group with mental health professionals provided input for further improvement of the intervention. We designed a new prototype of the intervention with visual support by the illustrator with lived experience in mental health. We presented this modified intervention to nineteen experts across the country. In the recruitment of these experts we combined a targeted sample with snowball sampling. There was no fixed list with all the experts we wanted to include at the beginning; we were open to suggestions from the experts we met on including additional experts. The final group of experts consisted of professionals with as many different backgrounds and opinions as possible, including nine experts by experience, five of whom had intellectual disabilities (Supplementary material 1).

We held the first semi-structured interviews in January 2020, using a topic list (Supplementary material 2). The interviews lasted 1 hour on average and we conducted them face to face until the Covid-19 pandemic started. From March 2020 we conducted interviews online. Three experts by experience of MID/BIF were interviewed by their supervisors at the affiliated university, because these experts needed more time and guidance. The experts had knowledge about recovery in mental health, about MID/BIF or both, and we asked them to evaluate the intervention drawing on their own expertise. We aimed to receive targeted feedback on the content, assignment, use of language and visual support, as detailed as possible and if possible with suggestions for improvement and further development. The experts indicated valuable points for improvement. We used insights that emerged during the interviews immediately, to make adjustments to the prototype as quickly as possible. For example, one of the expert pointed us to the Dutch guidelines for accessible or easy language “Taal voor Allemaal” (“Language for All”) that was being developed at the time. From then on, we have applied these principles as much as possible.

#### Cycle 3

In Cycle 3, we showed the revised prototype of the intervention to clients with SMHP and MID/BIF. Clients completing a different (generic) recovery program, similar to illness management and recovery (IMR) (55), were invited to review the new prototype of the intervention targeted at people with MID/BIF. These clients had already followed a 1 year recovery group training program in our organization (selected sample) and, therefore, they had some “recovery experience.” We asked these clients to review the new intervention in a focus group and give their opinion on this intervention chapter by chapter. We organized two focus groups to gather general opinions and impressions from clients before presenting the prototype to individual clients. In addition to the four main themes, these clients paid special attention to user-friendliness, attractiveness, clarity of the pictures, and the exercises. After the focus groups, the research team reconvened for reflection and to make adjustments.

#### Cycle 4

All mental health professionals in the eight FACT teams either received an e-mail with an invitation to join the study with a client from their own caseload or were approached by us personally for participation. We asked them to use the latest prototype of the intervention in the individual client’s treatment, with the client’s consent, from June 2021 until April 2022. In this cycle, we used self-assessment forms (Supplementary material 3) to collect the data. In this round, we examined the experiences and opinions of the clients and professionals with regard to the four main themes, for each chapter (sections in the intervention). There was a separate form for the professional. The professional helped the client with reading or filling out the form if necessary. Additionally, we asked professionals how many sessions they needed for the intervention, whether the manual was helpful, what worked well and what issues they encountered, points for improvement, and what the intervention could add to their treatment. The last question at the professionals was whether the intervention also provided input for a treatment or crisis plan.

### Data collection and evaluation

Prior to the first cycle, we collected topics and objectives from the relevant literature in order to shape the first prototype of the intervention and to use as categories for our coding tree. In cycle two and three, we collected our data through focus groups, interviews, and in cycle four we used fill-in forms. Focus groups and interviews were recorded with consent, written out verbatim and sent to the participants (only the professionals and experts) to increase credibility. We asked participants for their approval and response. All respondents gave their approval, and six gave a response. During the fourth cycle we used self-assessment forms (Supplementary Material 3). Professionals and clients answered questions after each chapter about the four main themes. There was also space for extra comments and notes. Content analysis in MAXQDA (56) was used to label the transcripts collected during focus groups, interviews and fill in forms, based on results after each cycle. The researchers labeled the data and classified it into the categories of content (topics, but also layout and structure), assignments, language use, and visual support. Between cycles, the research team got together for reflection and adjustments. We recorded the broad outline of the research, doubts, discussions, considerations, adjustments, and decisions made in a logbook. In case of conflict, the opinion of clients was decisive (Supplementary material 4). We recorded the details in meeting minutes.

## Results

Four rounds of data collection were followed by reflections on the findings of each individual cycle and modifications to the intervention. We will present the results per cycle, and the new insights and modifications based on those results. The details of the results are presented in more detail in Supplementary material 5.

### Cycle 1, mental health care professionals

The feedback in this round indicated several missing topics, exercises that required adjustments and the need for more drawings, for example about connectedness:

*“And I would also like to see an image of a person, […], but in the midst of other people. Also so that you portray that you are not alone.”*

The layout of the prototype required substantial adjustments: a larger font, more writing space, more colour and a clearer structure and more visuals. Nevertheless, all participants considered it a good starting document. Participants considered a manual for professionals as helpful, though the manual should not be too prescriptive.

#### New insights, reflection, and modifications

After the first focus group, we approached an illustrator with lived experience of mental health problems to make simple and clear pictures. Also, a list of themes for pictures emerged, for example recovery not as a straight line, the stages of recovery, and connectedness. We added missing topics, such as more attention to social relationships, addiction, and balancing stress/vulnerability and personal strengths. We did not include the missing topic of assertive behaviour, because modules on this already exist (57).

We added exercises such as a doll with two faces: one side highlighting experienced mental health problems and limitations and one side highlighting talents, positive qualities, and hobbies. We kept the true-or-false questions for the next cycle and clarified the explanations of assignments. We adopted the suggestion to add a scoring scale with emoticons/faces to assess the severity of psychological symptoms. Regarding language, the short sentences remained, but the many questions asked in succession were deleted. All layout changes were implemented. We added a question about a manual to the interview questions for the experts.

### Cycle 2, experts

The experts had different backgrounds, e.g., experts by experience mental health or intellectual disabilities, psychiatrists, psychologists, social work.

In terms of content, missed topics were: a client’s introduction to who you are; and adequate/inadequate coping strategies.

*“I think there is a need to look more extensively at what coping strategies people have. Withdrawing or, on the contrary, entering into unlimited contacts and calling everyone.”*

The experts provided comments and areas for improvement in the exercises, ranging from an exercise that many experts found too difficult (the puppet with the two sides) to an exercise that did not reveal any points for improvement (“This helps me in my recovery”). Experts in the field of inclusive language made the following suggestions: start each sentence on a new line, do not use questions in the running text, do not use commas, and do not use connecting words that make a sentence longer. Experts also pointed to too much nuance and duplication. Almost all experts who did not have a background of first-hand discussed about how to talk about “psychological complaints.” Experts by experience themselves were not concerned about the precise choice of words, finding “psychological complaints” to be the clearest. Sometimes it was important to use different words, for example “move on with your life” instead of “live beyond the illness.”

In general, experts liked the pictures but found some to be very complicated. Experts by experience of intellectual disabilities were critical of the facial expressions. All experts found the intervention of added value for treatment.

#### New insights, reflection, and modifications

As we spoke to more experts, the text also became longer and more nuanced. Two experts warned against too much text and nuance, and recommended deleting entire sections of explanation. During the reflection we concluded that we were too eager to please everyone. We therefore followed the advice and shortened the text.

The drawing of the puppet with two sides – problems and strengths – was deleted because it was too abstract and complicated.

A 10-point scale was replaced by a 5-point scale with five faces, a visual analogue scale (VAS), from very happy to very sad. The closing questions were replaced by questions such as “What do you want to remember for the future?” One expert advised us to add the question: “What would the artist have wanted to express with this picture?” when we presented the prototype to the client, to become clear whether they really understand the pictures.

### Cycle 3, clients with SMHP and mid/BIF with “recovery experience”

**Figure 2:**
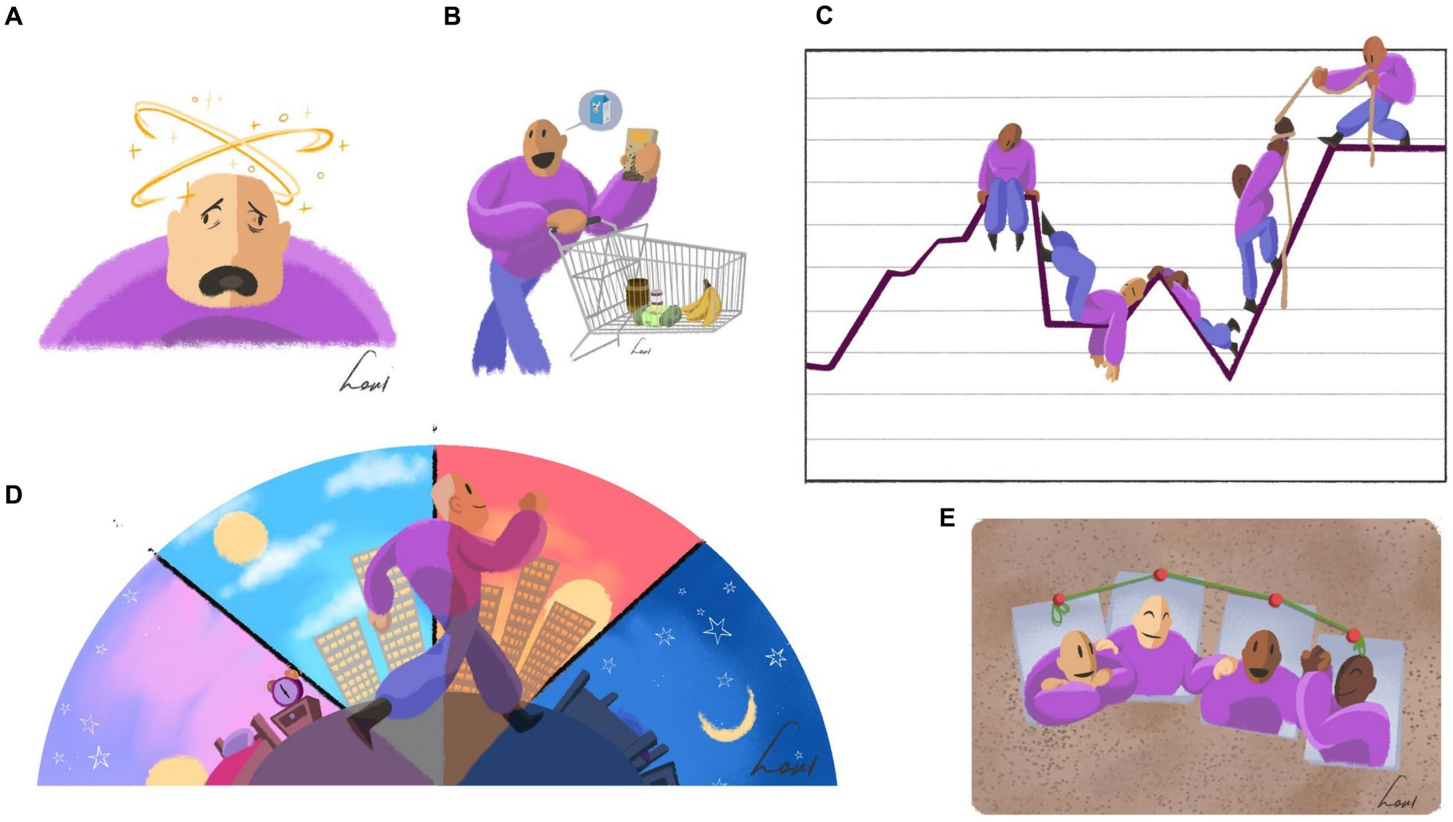
Collage of drawings that were positively received. **(A)** Your problems are in charge; **(B)** Carrying on with your life; **(C)** Recovery is an up-and-down process; **(D)** Day–night rhythm; **(E)** Recovery works better together.

The clients already knew each other and therefore probably dared to speak more freely. In addition to the four main themes, they paid special attention to user-friendliness, attractiveness, clarity of the pictures, and the exercises. As examples of inadequate coping we mentioned drinking alcohol, using drugs, and isolating yourself. All clients missed other subjects, such as take too much medication, not using medication, too much smoking, eating more, and stopping eating. All clients were positive about the introductory assignment “Tell us who you are, what you like and what you are good at.” Mentioning their own strengths is difficult for all clients. They experienced all other assignments as useful and clear. Clients had no problems with the choice of the wording “psychological complaints or problems” and they wondered what else to call it. They unanimously shared the opinion that the text was generally clear and easy to understand. In general, clients sometimes found some of the pictures very clear, did not understand others (e.g., a balancing puppet with two weights in his hands with the words “strength” and “complaint”; clients’ first thoughts were that he was a bodybuilder). They also gave comments and suggestions for improvement for other pictures. A recurring theme was the puppet’s facial expression, where the criticism was often that the puppet had a grumpy facial expression. An example of a picture that was immediately understood is the picture belonging to Stage 1 of recovery: “being overwhelmed by the condition,” or in easy language, “your complaints are in charge” (Figure 2):

*“As if he* (the puppet) *is a bit confused” and “that he has to take in too many things, because he has too many things in his head.”*

#### New insights, reflection, and modifications

Generally, clients critically evaluated the intervention and mentioned many points for improvement, including very detailed (formatting) points. We adopted improvements regarding formatting and layout and added, or deleted many spaces, dots, writing lines, and check boxes. We also adopted the recommendations in terms of content and added the missing topic of “inadequate coping.” In addition, we rewrote the explanation of fulfilling social roles in one’s life, and adapted the types of recovery to make them more structured and organized.

Clients generally evaluated the assignments positively. Only the assignment about strengths was not immediately clear. We added the suggestion that people could discuss this with each other or with other people who are important to them. The unclear sentences and words were changed. For example, the words “clumsy coping” has been changed to “not helpful coping,” and the words “social roles” were changed to “social contacts.” Most of the comments concerned the pictures. In total, three pictures were removed completely and three new ones were added, including another new picture in the “getting involved again” section. The facial expressions of certain pictures were changed. This modified prototype was used by participants in the next cycle.

### Cycle 4, individual mental health professionals and their clients

Clients found it important to talk about recovery and appreciated the attention paid to both complaints and strengths, although they all found it difficult to name their own strengths. The chapter “recovery takes time” was relevant but people also found it confronting. They found it interesting because you could see the stage they were in at that given time. When they came to the end of reviewing the prototype, in the recovery plan section, they were happy to see steps they could take in the future. In general, clients found the assignments fun and meaningful. They varied in their views of the writing assignments, most found them fine but a few found them a lot of work and no fun. They perceived the multiple choice checklist as nice and “thinking tasks” as difficult but also important. Clients understood the language well, and it was clear what was expected of them. Some words needed more explanation, especially for clients with Dutch as a second language. Professionals automatically explained difficult words themselves. While not everyone understood all the drawings, the majority of participants found the drawings fun, helpful and clear except for minor areas of improvement. However, for a minority of the clients, the drawings had no added value at all and they paid no attention to them. Some clients missed a drawing in the chapter about strengths. Clients unanimously responded positively to the VAS (faces scale).

Professionals unanimously agreed that this intervention was a nice method with clear explanations, a positive approach, and appropriate for positive psychiatry. It helped professionals to give substance to a recovery-oriented conversation and helped the client to gain more insight into themselves. Professionals expressed positive views on the topics covered:

*“Themes that are considered self-evident do not normally always come up in a treatment. Using this intervention make sure they do.”*

Professionals gave several points for improvement, including explaining the same thing twice and page layout, and they suggested a digital version. All professionals felt that the method helps increase the client’s understanding, and that the professional mental health workers can use the outcomes of the intervention for the treatment and crisis plan.

#### New insights, reflection, and modifications

We expanded the chapter on the topic of “strengths” because it was quite small compared to other chapters, while everyone (both clients and professionals) found it an important chapter. We added a list of examples of strengths, that clients can check if desired. The chapter “Types of Recovery” was large and confusing, and needed more structure. Possibly the use of sections and adjustments to the layout could improve this. The recovery plan section asked “What do you need to achieve that goal?” We provided the question “I need this” with additional explanation (e.g., “Think for example of people who can support you in this” or “What are your own strengths that you can use here?”). Clients did not understand all the drawings immediately. We decided to give the drawings titles, in the hope of improving understanding and opening up the conversation about the drawings a little more. Also, we added a drawing in the chapter of “Strengths.” Clients appreciated the VAS, which was in line with the response of the focus group in Cycle 3.

The final prototype contains the following chapters: introducing yourself, problems, strengths, recovery, recovery takes time, types of recovery, recovery plan and the last chapter, coming to a close. All chapters alternate comprehensible text with illustrations (visual support) and assignments. The different prototypes had led to the intervention “Routes to Recovery” (Supplementary material 6, an English translation of the intervention). The intervention is only investigated in Dutch language.

## Discussion

This article outlines the development of a recovery-oriented intervention in mental health care for people with both SMHP and MID/BIF, which provides guidance and a concrete tool for professionals. This intervention aims to encourage professionals to engage with their clients with SMHP and MID/BIF and discuss all facets of recovery in order to provide tailored services to these clients. The study demonstrated that clients felt it was important to talk about recovery and the steps they can take in the future, but had difficulty naming their own strengths. Throughout the study, clients gave many helpful suggestions for improving the intervention. Professionals experienced the intervention as a positive approach that gave substance to a recovery-oriented conversation and thus provided the client with greater insight into their own recovery. According to the feedback from mental health professionals, they can use the intervention to complement the usual treatment and readily available methods. Outcomes from the intervention that provide insight into complaints, inadequate coping, strengths, and things to do that help in recovery can be used as input for the crisis plan. Outcomes that include recovery goals, as mentioned in the recovery plan, can be incorporated in a treatment plan, as used in regular mental health care. Interestingly, some professionals said that the intervention was especially useful at the beginning of treatment, when formulating goals, while others seemed to find this intervention useful at the end of treatment, to see if there were any outstanding goals. Professionals and clients provided feedback on four main themes: content, assignments, understandable language, and visual support (drawings).

In terms of content, clients felt it was important to talk about mental health problems they were experiencing, and they were able to name these easily. They could not name their own strengths and often needed help. This is consistent with a study in which young people with disabilities rated themselves lower on each character strength than young people without disabilities (58). In disability care, limited but increasing attention has been paid to personal strengths and positive psychology (58–60). One study of the character strengths of people with disabilities used an international dataset from the via Institute on Character. It indicated that the top five character strength scores were love of learning, honesty, fairness, judgment, and appreciation of beauty and excellence (59). Clients also felt it was important to talk about steps they can take in the future in the “Recovery plan” chapter. Promoting self-determination and opportunities for people with intellectual disabilities to make their own choices has become a best practice (61). People with ID indicate that living a self-determined life means making choices and working towards goals (37). It starts with person-centered planning to identify what is important to a person, and a focus on the individual’s dreams, personal preferences, and interests (62).

In the earlier cycles of the current study, participants identified many points for improvement in the designed assignments. Consequently by the fourth cycle, the pilot, the exercises had been developed further and they received mostly positive feedback. All clients understood the visual analogue scale (VAS; five faces) well, which we used to score the burden of psychological symptoms, and the satisfaction with their own strengths. This is in line with recent research (63) indicating that there is moderate confidence that VAS scales can produce reliable and meaningful results in people with MID/BIF to depict pain and emoticons, although we used it for mental burden and satisfaction with strengths. While open-ended questions can be used to ask for opinions (63), the question “What are your strengths?” was too difficult in terms of its content as we mentioned before. We hope to have solved this problem by adding the suggestion to ask for support (34) from significant others and to think about what the person used to be good at, before his/her mental health problems emerged. This latter adaptation fits into a solution-focused approach, in which asking about and exploring times when the problem was less severe is an integral part; this has also been investigated for people with ID (64). The “yes/no” questions in the section on rhythm and regularity were also felt by clients to be useful and clear, and had space for comments. There is high confidence that this type of question is understood by people with MID/BIF (63).

The use of understandable language was already a focus when writing the first prototype, in the sense of aiming to use short sentences, as few difficult words and as little abstract language as possible. Nevertheless, as the project progressed, we encountered additional requirements and rules for making language as understandable as possible. We adopted the Dutch guidelines for accessible or easy language “Taal voor Allemaal” (“Language for All”), and asked experts in the field to review our text. Although the adjustment of language also worked well in other research (65), just adapting the language does not suffice to increase understanding. A later review (66) demonstrated that individually tailored information is likely to better meet the personal health information needs of people with ID. This likely means for this recovery intervention that professionals will sometimes need to help their clients translate the information provided in the intervention to fit their own personal situation.

Finally, our clients varied in their opinions about the use of drawings for visual support. This is consistent with research showing that clients also have a preference for the type of images used, demonstrating that it is partly a matter of personal taste (67). In our intervention we used illustrations; illustrations are more symbolic and convey more general messages (68). This may explain why not everyone understood all the pictures. Although we showed the pictures to people with SMHP and MID/BIF in a pilot test (63), they did not improve the understanding for all participants. Clients rated the pictures as fun and sometimes helpful, but it remains unclear whether the pictures helped them understand the text. Pictures accompanied by an explanation did seem to convey the intended meaning better (69). Therefore, we added titles to the illustrations after the pilot study. It is the combination of text and illustrations that provides understandable information (67).

The United Nations Convention on the Rights of Persons with Disabilities states that persons with disabilities have the same rights as everyone else (70). People with intellectual disabilities should therefore have the same rights to appropriate mental health care treatment, even if it means making adjustments to make it accessible for people with intellectual disabilities. This may be where a next task for mental health care providers lies: setting structural adaption of treatment in motion and taking down unnecessary barriers, such as inaccessible language. A recent report in Netherlands pointed out that more attention is needed for participation of people with intellectual disabilities. A worrying trend in recent years is that people with intellectual disabilities almost always have the lowest level of participation (71). People with both intellectual disabilities and SMHP are likely to experience even more problems with participation. We hope that engaging in a conversation about recovery, including rediscovering strengths, also contributes to societal recovery of clients with both SMHP and MID/BIF.

### Strengths and limitations

To our knowledge, this is the first conversation intervention on recovery for people with SMHP and MID/BIF that has been developed with the help of professionals, experts by experience and other experts, and with the help of clients receiving treatment from the mental health services. To increase reliability, researchers from different backgrounds and perspectives were included in the research team to promote research triangulation. The long development period of nearly 4 years allowed the inclusion of many different participants and perspectives. Although no person with an MID/BIF participated in the research team for the entire research cycle (which is considered as a best practice), the active involvement of people with an MID/BIF was central to this study and added value (72). In Cycle 4, the intervention was administered to the client by the client’s own professional, rather than by the researchers, to avoid socially desirable responses and to gather opinions from clients and professionals. Piloting the intervention gave us a more realistic idea about the usage of the intervention in daily practice. There is little to say about the duration of the intervention, as this was customized to suit the client. Some took three sessions and others twelve. This can be explained by the large differences between clients in terms of cognitive ability, degree of concentration, whether Dutch is the mother tongue, and possibly other factors. For some clients it can be appropriate to use the intervention at the beginning of the treatment, as an introduction and to set goals, but for clients who have been in treatment for a long time it can also be a way to see what stage they are at in terms of treatment and recovery.

Several limitations regarding this study should be mentioned. First, we investigated clients with MID or BIF as one single group (MID/BIF). This MID/BIF label is often used in Netherlands, while differences also exist within this group in their personal and environmental characteristics (73). Also, the international literature often deals either with the MID group or the BIF group. Second, a limitation is that we did not have someone with intellectual disability in our research team, helping us to write the intervention (the booklet) and conduct the study. This could have improved our project. Third, we aimed to provide guidance to all professionals to help them start a conversation about recovery with their clients with both SMHP and MID/BIF. Unfortunately, professionals who have no affinity with the MID/BIF target group hardly participated in the study, and therefore it is not possible to say how they perceive this intervention. Only one of the fifteen participating professionals in the pilot round had no affinity at all, and two had limited affinity with the target group. Given that MID/BIF is common among clients with SMHP, this intervention may well be particularly necessary, for professionals who currently say they have no affinity with the target group. Finally, we did not test the manual “Points to consider in conversation, with a workbook on recovery.” Experts recommended this manual, but unfortunately in Cycle 4, professionals thought the question was about the intervention rather than the manual. Titles accompanying the pictures, to support understanding, were added after Cycle 4 and were therefor also not examined. As a result, we lack information about the added value of the manual and the pictures, and possible areas for improvement.

### Conclusion

Developing the intervention “Routes to Recovery” (Supplementary material 6, translated from Dutch) is a first step in supporting clients with SMHP and MID/BIF and professionals to engage in a conversation about personal strengths and what the client needs in his/her recovery process. A crucial finding was that clients could easily name their mental health problems, but had difficulty acknowledging their own strengths, and often needed help in doing so. This intervention incorporates understandable language about all facets of recovery, with illustrations and exercises. Routes to Recovery complements other regular forms of care, and the outcomes can be incorporated in a treatment and crisis plan. This makes it a potentially useful and usable method for everyday professional practice, in a variety of mental health care settings. Future research is needed to investigate the effectiveness of the intervention as well as the process by which the implementation can be implemented in routine.

## Data availability statement

The original contributions presented in the study are included in the article/Supplementary material, further inquiries can be directed to the corresponding author.

## Ethics statement

The studies involving human participants were reviewed and approved by The medical ethic commission (METC) Leiden Den Haag Delft. The patients/participants provided their written informed consent to participate in this study.

## Author contributions

IB was the initiator, principal investigator, a member of the research team, and wrote the article. AB, ME, LJ, and MH were all members of the research team. JW and LM provided advice on the study design and commented on the article. All authors contributed to the article and approved the submitted version.

## Conflict of interest

The authors declare that the research was conducted in the absence of any commercial or financial relationships that could be construed as a potential conflict of interest.

## Publisher’s note

All claims expressed in this article are solely those of the authors and do not necessarily represent those of their affiliated organizations, or those of the publisher, the editors and the reviewers. Any product that may be evaluated in this article, or claim that may be made by its manufacturer, is not guaranteed or endorsed by the publisher.

## Supplementary material

The Supplementary material for this article can be found online at: https://www.frontiersin.org/articles/10.3389/fpsyt.2023.1184798/full#supplementary-material

## References

[1] Nieuwenhuis JG Lepping P Mulder NL Nijman HLI Veereschild M Noorthoorn EO. Increased prevalence of intellectual disabilities in higher-intensity mental healthcare settings. BJPsych Open. (2021) 7:e83. doi: 10.1192/bjo.2021.28, PMID: 33883055 PMC8086388

[2] van Esch AYM de Vries J Masthoff EDM. Screening for intellectual disability in Dutch psychiatrically disturbed detainees: assessing the psychometric properties of the screener for intelligence and learning disability (SCIL). J Appl Res Intellect Disabil. (2020) 33:1418–27. doi: 10.1111/jar.12769, PMID: 32578391 PMC7687161

[3] Nieuwenhuis JG Noorthoorn EO Nijman HL Naarding P Mulder CL. A blind spot? Screening for mild intellectual disability and borderline intellectual functioning in admitted psychiatric patients: prevalence and associations with coercive measures. PLoS One. (2017) 12:e0168847. doi: 10.1371/journal.pone.0168847, PMID: 28151977 PMC5289434

[4] Smits HJH Seelen-de Lang BL Nijman HLI Penterman EJM Nieuwenhuis JG Noorthoorn EO. The predictive value of mild intellectual disability/borderline intellectual functioning and PTSD for treatment results in severely mentally ill patients. Tijdschr Psychiatr. (2020) 62:868–77. PMID: 33184818

[5] Nouwens PJG Smulders NBM Embregts P van Nieuwenhuizen C. Meeting the support needs of persons with mild intellectual disability or borderline intellectual functioning: still a long way to go. J Intellect Disabil Res. (2017) 61:1104–16. doi: 10.1111/jir.12427, PMID: 29047184

[6] Parabiaghi A Bonetto C Ruggeri M Lasalvia A Leese M. Severe and persistent mental illness: a useful definition for prioritizing community-based mental health service interventions. Soc Psychiatry Psychiatr Epidemiol. (2006) 41:457–63. doi: 10.1007/s00127-006-0048-0, PMID: 16565917

[7] Delespaul PH de consensusgroep EPA. Consensus regarding the definition of persons with severe mental illness and the number of such persons in Netherlands. Tijdschr Psychiatr. (2013) 55:427–38. PMID: 23864410

[8] Anthony WA. Recovery from mental illness: the guiding vision of the mental health service system in the 1990s. Psychosocial Rehabilitation J. (1993) 16:11–23. doi: 10.1037/h0095655

[9] Davidson L. Considering recovery as a process: or, life is not an outcome. A. Rudnicj ed. Recovery of people with mental illness. Phil Relat Persp. New York: Oxford University Press. (2012):252–63. doi: 10.1093/med/9780199691319.003.0016

[10] Leamy M Bird V Le Boutillier C Williams J Slade M. Conceptual framework for personal recovery in mental health: systematic review and narrative synthesis. Br J Psychiatry. (2011) 199:445–52. doi: 10.1192/bjp.bp.110.083733, PMID: 22130746

[11] van Weeghel J van Zelst C Boertien D Hasson-Ohayon I. Conceptualizations, assessments, and implications of personal recovery in mental illness: a scoping review of systematic reviews and meta-analyses. Psychiatr Rehabil J. (2019) 42:169–81. doi: 10.1037/prj0000356, PMID: 30843721

[12] van Os J Guloksuz S Vijn TW Hafkenscheid A Delespaul P. The evidence-based group-level symptom-reduction model as the organizing principle for mental health care: time for change? World Psychiatry. (2019) 18:88–96. doi: 10.1002/wps.20609, PMID: 30600612 PMC6313681

[13] Lloyd C Waghorn G Williams PL. Conceptualising recovery in mental health rehabilitation. Br J Occup Ther. (2008) 71:321–8. doi: 10.1177/030802260807100804

[14] van Aken BC Bakia A Wierdsma AI Voskes Y van Weeghel J van Bussel EMM. UP'S: a cohort study on recovery in psychotic disorder patients: design protocol. Front Psych. (2021) 11:609530. doi: 10.3389/fpsyt.2020.609530, PMID: 33584375 PMC7874019

[15] Stuart SR Tansey L Quayle E. What we talk about when we talk about recovery: a systematic review and best-fit framework synthesis of qualitative literature. J Ment Health. (2017) 26:291–304. doi: 10.1080/09638237.2016.1222056, PMID: 27649767

[16] Boevink W. Life beyond psychiatry. A. Rudnicj ed. Recovery of people with mental illness. Phil Rel Persp. New York: Oxford University Press. (2012):15–29. doi: 10.1093/med/9780199691319.003.0002

[17] Chen W Epstein A Toner M Murphy N Rudaizky D Downs J. Enabling successful life engagement in young people with ADHD: new components beyond adult models of recovery. Disabil Rehabil. (2022) 45:2288–300. doi: 10.1080/09638288.2022.2087763 35944517

[18] Mueser KT Corrigan PW Hilton DW Tanzman B Schaub A Gingerich S. Illness management and recovery: a review of the research. Psychiatr Serv. (2002) 53:1272–84. doi: 10.1176/appi.ps.53.10.1272 12364675

[19] Cook JA Copeland ME Jonikas JA Hamilton MM Razzano LA Grey DD. Results of a randomized controlled trial of mental illness self-management using wellness recovery action planning. Schizophr Bull. (2012) 38:881–91. doi: 10.1093/schbul/sbr012, PMID: 21402724 PMC3406522

[20] Fukui S Starnino VR Susana M Davidson LJ Cook K Rapp CA. Effect of wellness recovery action plan (WRAP) participation on psychiatric symptoms, sense of hope, and recovery. Psychiatr Rehabil J. (2011) 34:214–22. doi: 10.2975/34.3.2011.214.222, PMID: 21208860

[21] Gestel-Timmermans V JAWM B van Nieuwenhuizen C. Effects of a peer-run course on the recovery of people with major psychiatric problems: a randomised controlled trial. Recovery is up to you: evaluation of a peer-run course Psychiatric Serv. (2012) 63: 54–60. doi: 10.1176/appi.ps.201000450 22227760

[22] Boevink W Kroon H van Vugt M Delespaul P van Os J. A user-developed, user run recovery programme for people with severe mental illness: a randomised control trial. Psychosis. (2016) 8:287–300. doi: 10.1080/17522439.2016.1172335

[23] APA. Handboek voor de classificatie van psychische stoornissen. DSM-5. Amsterdam: Uitgeverij Boom; (2014)

[24] Hassiotis A Strydom A Hall I Ali A Lawrence-Smith G Meltzer H. Psychiatric morbidity and social functioning among adults with borderline intelligence living in private households. J Intellect Disabil Res. (2008) 52:95–106. doi: 10.1111/j.1365-2788.2007.01001.x, PMID: 18197948

[25] Seltzer MM Floyd F Greenberg J Lounds J Lindstromm M Hong J. Life course impacts of mild intellectual deficits. Am J Ment Retard. (2005) 110:451–68. doi: 10.1352/0895-8017(2005)110[451:LCIOMI]2.0.CO;2, PMID: 16212448

[26] Westerinen H Kaski M Virta L Almqvist F Iivanainen M. Prevalence of intellectual disability: a comprehensive study based on national registers. J Intellect Disabil Res. (2007) 51:715–25. doi: 10.1111/j.1365-2788.2007.00970.x, PMID: 17845240

[27] Cuypers M Tobi H Naaldenberg J Leusink GL. Linking national public services data to estimate the prevalence of intellectual disabilities in Netherlands: results from an explorative population-based study. Public Health. (2021) 195:83–8. doi: 10.1016/j.puhe.2021.04.002, PMID: 34062276

[28] Hassiotis A Ukoumunne O Tyrer P Piachaud J Gilvarry C Harvey K. Prevalence and characteristics of patients with severe mental illness and borderline intellectual functioning. Report from the UK700 randomised controlled trial of case management. Br J Psychiatry. (1999) 175:135–40. doi: 10.1192/bjp.175.2.135, PMID: 10627795

[29] Seelen-de Lang BL Smits HJH Penterman BJM Noorthoorn EO Nieuwenhuis JG Nijman HLI. Screening for intellectual disabilities and borderline intelligence in Dutch outpatients with severe mental illness. J Appl Res Intellect Disabil. (2019) 32:1096–102. doi: 10.1111/jar.12599, PMID: 31033102

[30] Stuurman S Mulder A Van Straaten B Kruijt P De Baan M Mulder N. Intelligentieonderzoek bij patiënten van assertive community treatment (ACT)-teams [a study into intelligence of patients of assertive community treatment (ACT)—teams]. Rotterdam (NL) Bavo Europoort (2008)

[31] Mazza MG Rossetti A Crespi G Clerici M. Prevalence of co-occurring psychiatric disorders in adults and adolescents with intellectual disability: a systematic review and meta-analysis. J Appl Res Intellect Disabil. (2020) 33:126–38. doi: 10.1111/jar.12654, PMID: 31430018

[32] van Duijvenbode N VanDerNagel JE Didden R Engels RC Buitelaar JK Kiewik M. Substance use disorders in individuals with mild to borderline intellectual disability: current status and future directions. Res Dev Disabil. (2015) 38:319–28. doi: 10.1016/j.ridd.2014.12.029, PMID: 25577182

[33] Hartley SL MacLean WE. Perceptions of stress and coping strategies among adults with mild mental retardation: insight into psychological distress. Am J Ment Retard. (2005) 110:285–97. doi: 10.1352/0895-8017(2005)110[285:POSACS]2.0.CO;2, PMID: 15941365

[34] Snell ME Luckasson R Borthwick-Duffy WS Bradley V Buntinx WH Coulter DL. Characteristics and needs of people with intellectual disability who have higher IQs. Intellect Dev Disabil. (2009) 47:220–33. doi: 10.1352/1934-9556-47.3.220, PMID: 19489667

[35] Allen D Langthorne P Tonge B Emerson E McGill P Fletcher R. Towards the prevention of behavioural and psychiatric disorders in people with intellectual disabilities. J Appl Res Intellect Disabil. (2013) 26:501–14. doi: 10.1111/jar.12050, PMID: 23712642

[36] Nouwens PJ Lucas R Embregts PJ van Nieuwenhuizen C. In plain sight but still invisible: a structured case analysis of people with mild intellectual disability or borderline intellectual functioning. J Intellect Develop Disabil. (2017) 42:36–44. doi: 10.3109/13668250.2016.1178220

[37] Shogren KA Broussard R. Exploring the perceptions of self-determination of individuals with intellectual disability. Intellect Dev Disabil. (2011) 49:86–102. doi: 10.1352/1934-9556-49.2.86, PMID: 21446872

[38] Morisse F Vandemaele E Claes C Claes L Vandevelde S. Quality of life in persons with intellectual disabilities and mental health problems: an explorative study. ScientificWorldJournal. (2013) 2013:491918:1–8. doi: 10.1155/2013/491918 23476137 PMC3576796

[39] Moonen X. Easy language in Netherlands. Lindholm C VU, (Ed.) Handbook of easy languages of Europe. Berlin: Frank & Timme; (2021) p. 345–370.

[40] Berger I van der Hout M Hoogenboom A Berger E Mulder CL. Modifications in therapy for patients with severe mental illness and intellectual disability: a qualitative study. Tijdschr Psychiatr. (2019) 61:375–83. PMID: 31243747

[41] Martinelli TF Meerkerk GJ Nagelhout GE Brouwers EPM van Weeghel J Rabbers G. Language and stigmatization of individuals with mental health problems or substance addiction in Netherlands: an experimental vignette study. Health Soc Care Community. (2020) 28:1504–13. doi: 10.1111/hsc.12973, PMID: 32154632 PMC7496658

[42] Cooper SA van der Speck R. Epidemiology of mental ill health in adults with intellectual disabilities. Curr Opin Psychiatry. (2009) 22:431–6. doi: 10.1097/YCO.0b013e32832e2a1e 19535982

[43] Inperspectief RD. Gedragsproblemen, psychiatrische stoornissen en lichte verstandelijke beperking. Houten: Bohn Stafleu van Loghum (2006).

[44] Wieland J. Focus on borderline intellectual functioning in mental health care. Tijdschr Psychiatr. (2019) 61:761–5. PMID: 31907885

[45] Crowley V Rose J Smith J Hobster K Ansell E. Psycho-educational groups for people with a dual diagnosis of psychosis and mild intellectual disability: a preliminary study. J Intellect Disabil. (2008) 12:25–39. doi: 10.1177/1744629507086606, PMID: 18337299

[46] Raftery M Burke K Murray N O'Duinn O Murray I Hallahan B. An intensive personalised support approach to treating individuals with psychosis and co-morbid mild intellectual disability. Ir J Psychol Med. (2017) 34:99–109. doi: 10.1017/ipm.2016.19, PMID: 30115213

[47] Westerhof GJ Beernink J Sools A. Who am I? A life story intervention for persons with intellectual disability and psychiatric problems. Intellect Dev Disabil. (2016) 54:173–86. doi: 10.1352/1934-9556-54.3.173, PMID: 27268473

[48] van der Meer L Jonker T Wadman H Wunderink C van Weeghel J Pijnenborg GHM. Targeting personal recovery of people with complex mental health needs: the development of a psychosocial intervention through user-centered design. Front Psych. (2021) 12:635514. doi: 10.3389/fpsyt.2021.635514, PMID: 33897494 PMC8060492

[49] Van Lieshout FJG Cardiff S. Actie-onderzoek. Assen Koninklijke van Gorcum: Principes voor verandering in zorg en welzijn (2017).

[50] van Veldhuizen JR. FACT: a Dutch version of ACT. Community Ment Health J. (2007) 43:421–33. doi: 10.1007/s10597-007-9089-4, PMID: 17514502

[51] Westen K Boyle P Kroon H. An observational comparison of FACT and ACT in Netherlands and US. BMC Psychiatr. (2022) 22:311. doi: 10.1186/s12888-022-03927-x, PMID: 35505332 PMC9063161

[52] Rotterdam. Available at: https://wijkprofiel.rotterdam.nl/nl/2022/rotterdam.

[53] Available at: https://digitaal.scp.nl/.

[54] Lanting. Green Paper Bedrijfskundig Meesterschap: Denkmodel Actiegericht ontwikkelen (Business Mastery: Thinking Model Actionable Development). (2010)

[55] Roosenschoon BJ van Weeghel J Deen ML van Esveld EW Kamperman AM Mulder CL. Effects of illness management and recovery: a multicenter randomized controlled trial. Front Psych. (2021) 12:723435. doi: 10.3389/fpsyt.2021.723435, PMID: 34970161 PMC8712643

[56] MAXQDA. Available at: https://www.maxqda.com/mobile-ab-test-2023-05.

[57] Nezu CM Nezu AM Arean P. Assertiveness and problem-solving training for mildly mentally retarded persons with dual diagnoses. Res Dev Disabil. (1991) 12:371–86. doi: 10.1016/0891-4222(91)90033-O, PMID: 1792363

[58] Niemiec RM Shogren KA Wehmeyer ML. Character strengths and intellectual and developmental disability: a strengths-based approach from positive psychology. Educ Train Aut Dev Disabil. (2017) 52:13–25.

[59] Umucu E Lee B Genova HM Chopik WJ Sung C Yasuoka M. Character strengths across disabilities: an international exploratory study and implications for positive psychiatry and psychology. Front Psych. (2022) 13:863977. doi: 10.3389/fpsyt.2022.863977, PMID: 35280155 PMC8914428

[60] Wehmeyer ML. The future of positive psychology and disability. Front Psychol. (2021) 12:790506. doi: 10.3389/fpsyg.2021.790506, PMID: 34956017 PMC8696272

[61] Wehmeyer ML Abery BH. Self-determination and choice. Intellect Dev Disabil. (2013) 51:399–411. doi: 10.1352/1934-9556-51.5.399 24303826

[62] Thompson JR Bradley VJ Buntinx WH Schalock RL Shogren KA Snell ME. Conceptualizing supports and the support needs of people with intellectual disability. Intellect Dev Disabil. (2009) 47:135–46. doi: 10.1352/1934-9556-47.2.135, PMID: 19368481

[63] Kooijmans R Mercera G Langdon PE Moonen X. The adaptation of self-report measures to the needs of people with intellectual disabilities: A systematic review. Clin Psychol Sci Practice. (2022) 29:250–71. doi: 10.1037/cps0000058

[64] Roeden J Maaskant M Curfs L. Processes and effects of solution-focused brief therapy in people with intellectual disabilities: a controlled study. J Intellect Disabil Res. (2014) 58:307–20. doi: 10.1111/jir.12038, PMID: 23521046

[65] Karreman J Van der Geest T Buursink E. Accessible website content guidelines for users with intellectual disabilities. J Appl Res Intellect Disabil. (2007) 20:510–8. doi: 10.1111/j.1468-3148.2006.00353.x

[66] Chinn D Homeyard C. Easy read and accessible information for people with intellectual disabilities: is it worth it? A meta-narrative literature review. Health Expect. (2017) 20:1189–200. doi: 10.1111/hex.12520, PMID: 27862757 PMC5689240

[67] Waight M Oldreive W. Investigating accessible information formats with people who have learning disabilities. Learn Disabil Pract. (2022) 25:23–30. doi: 10.7748/ldp.2020.e2031

[68] Sutherland RJ Isherwood T. The evidence for easy-read for people with intellectual disabilities: a systematic literature review. J Pol Pract Intellect Disabil. (2016) 13:297–310. doi: 10.1111/jppi.12201

[69] Oldreive W Waight M. Enabling access to information by people with learning disabilities. Tizard Learn Disabil Rev. (2013) 18:5–15. doi: 10.1108/13595471311295950

[70] Convention On The Rights Of Persons With Disabilities (CRPD) (2016) Available at: https://social.desa.un.org/issues/disability/crpd/convention-on-the-rights-of-persons-with-disabilities-crpd. 10.1515/9783110208856.203 18348362

[71] Nivel. Doelgericht beleid blijft nodig om participatie van mensen met een beperking te verbeteren (2023) Available at: https://www.nivel.nl/nl/nieuws/doelgericht-beleid-blijft-nodig-om-participatie-van-mensen-met-een-beperking-te-verbeteren.

[72] Frankena TK Naaldenberg J Cardol M Linehan C van Schrojenstein Lantman-de Valk H. Active involvement of people with intellectual disabilities in health research – a structured literature review. Res Dev Disabil. (2015) 45–46:271–83. doi: 10.1016/j.ridd.2015.08.004, PMID: 26280692

[73] Nouwens PJG Lucas R Smulders NBM Embregts P van Nieuwenhuizen C. Identifying classes of persons with mild intellectual disability or borderline intellectual functioning: a latent class analysis. BMC Psychiatry. (2017) 17:257. doi: 10.1186/s12888-017-1426-8, PMID: 28716016 PMC5512980

