# Supplementary material for: Developing a recovery-oriented intervention for people with severe mental illness and an intellectual disability: design-oriented action research
Source: Front Psychiatry. 2023 Jul 19;14:1184798. doi: 10.3389/fpsyt.2023.1184798 (PMC10395094; doi:10.3389/fpsyt.2023.1184798)
Supplement: Supplementary file 1 [file Data_Sheet_1.PDF]

Antes PG

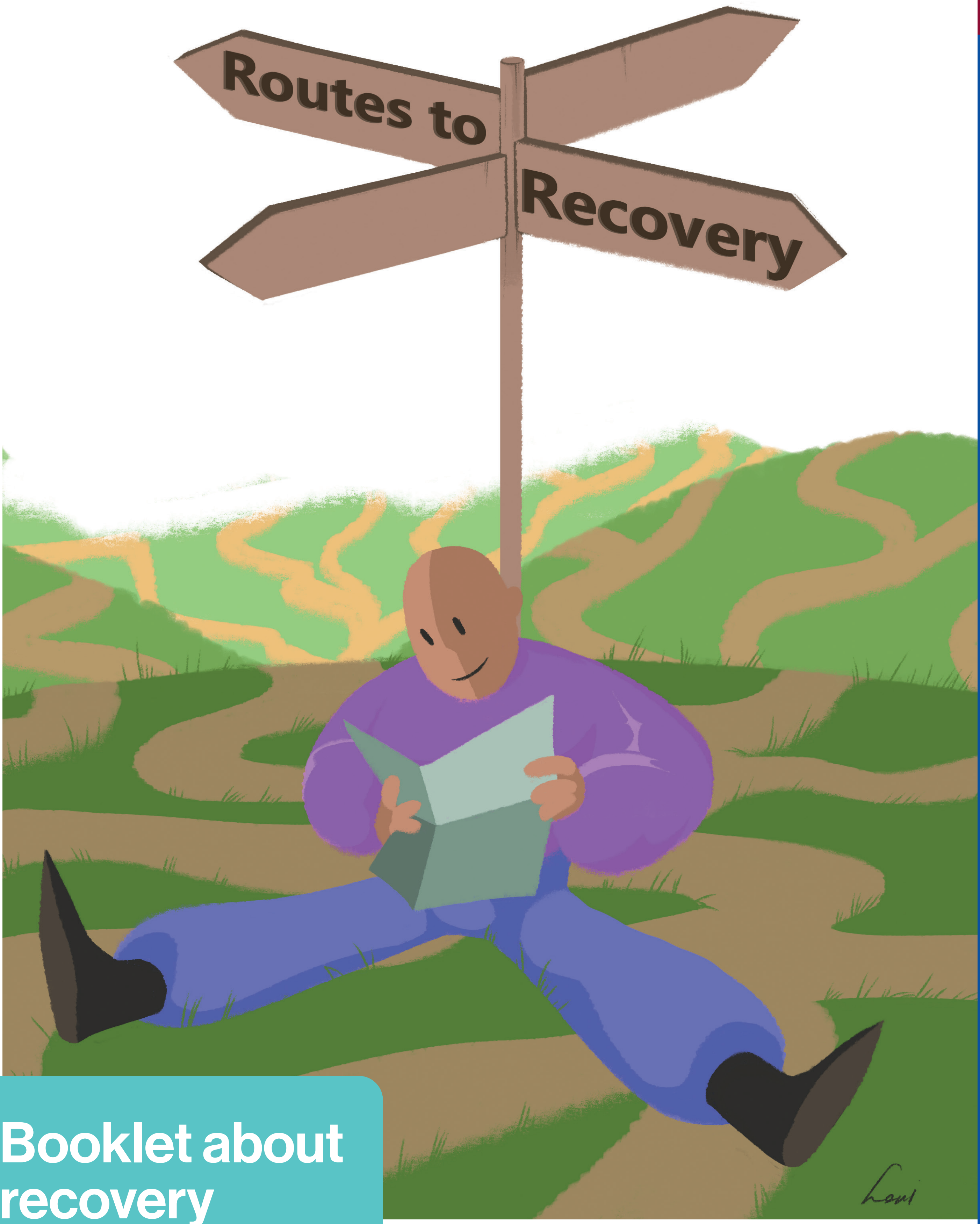

Booklet about recovery

Name : ....

This booklet was made by:
Ingeborg Berger (Nurse practitioner)
Margot van Ewijk (job coach, artist)
Anne Bruineberg (Expert by experience)

The illustrations are made by: Levi de Jong

*Note! This intervention has only been researched in Dutch.

Explanation

This booklet is about mental health problems.
You too may have mental health problems.
You can learn how to cope with them.
We sometimes call learning to cope with problems ‘recovery’.

You are going to use this booklet together.
You will work on goals together.
You can also decide on new goals.
You will learn new things about yourself
and about your mental health problems.
You will talk about what works for you.
You choose how fast or slowly you go through this booklet.

It might take eight sessions.
But you can take longer if you want.
The booklet has eight chapters.
Each chapter starts with an introduction.
Then you do an exercise.
You’ll see this picture next to each exercise.

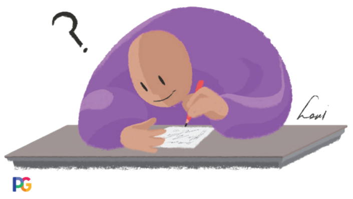

The next page gives an overview of the chapters.

Booklet contents

Chapter 1 Introducing yourself 6

Exercise 1 7

Chaptern 2 Problems 8

Exercise 2A 9

Exercise 2B 11

Exercise 2C 12

Chapter 3 Strengths 14

Exercise 3A 14

Exercise 3B 17

Chapter 4 Recovery 18

Exercise 4 19

Chapter 5 Recovery takes time 20

Exercise 5A 22

Exercise 5B 22

Chapter 6 Types of recovery 24

Exercise 6A 25

Exercise 6B 26

Exercise 6C 28

Exercise 6D 30

Chapter 7 Recovery plan 32

Exercise 7A 32

Exercise 7B 33

Exercise 7C 34

Chapter 8 Coming to a close 36

Exercise 8 36

Chapter 1 Introducing yourself

Everyone is different.

Tell us who you are, what you enjoy and what you are good at.

Exercise 1

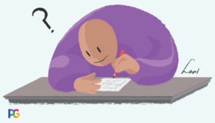

Name:

....

Age:

....

My favourite food:

....

My pets:

....

My favourite music:

....

My favourite film:

....

My hobbies:

....

This is what I'm good at:

....

....

....

This is what I find difficult :

....

....

....

This is what I enjoy:

....

....

....

My biggest wish is:

....

....

....

....

Chapter 2 Problems

Difficult periods are part of life.
So are feeling sad, angry or afraid.
People sometimes get mental health problems.
That's perfectly normal.
You too have mental health problems.
Here are some examples of mental health problems.

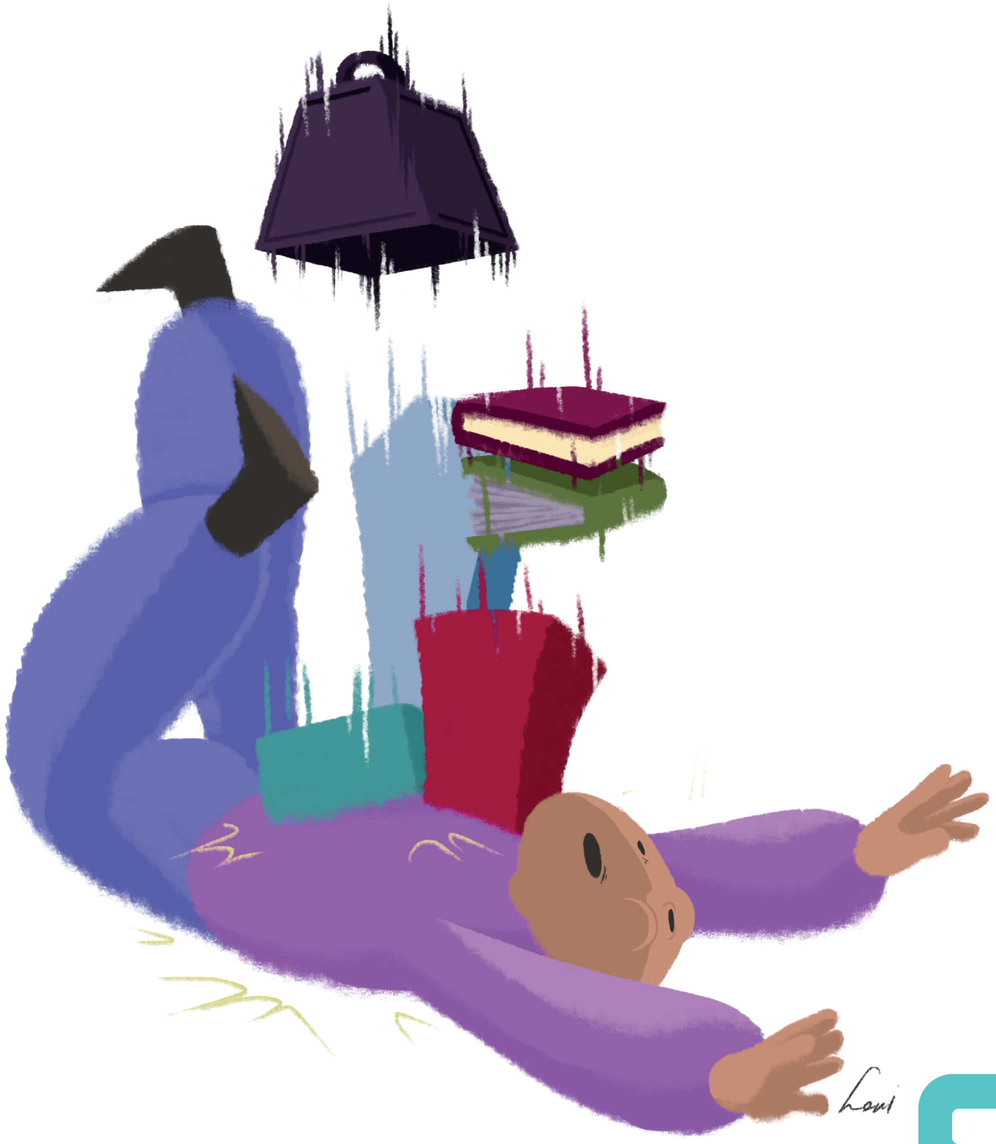

> Problems can weigh you down.

Exercise 2a

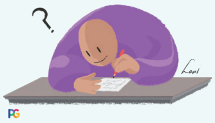

✓ **Tick the box if you too have had this problem:**

- ☐ Feeling overstimulated
- ☐ Thinking too much
- ☐ Sleeping poorly
- ☐ Feeling sad
- ☐ Feeling anxious
- ☐ Hearing voices
- ☐ Having difficulty trusting people
- ☐ Being hyperactive
- ☐ Feeling restless
- ☐ Feeling chaos in your head
- ☐ Feeling angry
- ☐ Eating lots, or eating almost nothing
- ☐ Being confused
- ☐ Thinking about horrible things that happened in the past

- ☐ ....
- ☐ ....
- ☐ ....
- ☐ ....
- ☐ ....
- ☐ ....

Everyone experiences their mental health problems differently.
Sometimes the problems can be serious.
Or you can have lots of problems at the same time.
Your problems might last a long time.
You might get into difficulties at school,
at your work or with other people in your life.

If you experience a lot of problems, it is a good idea to ask for help.

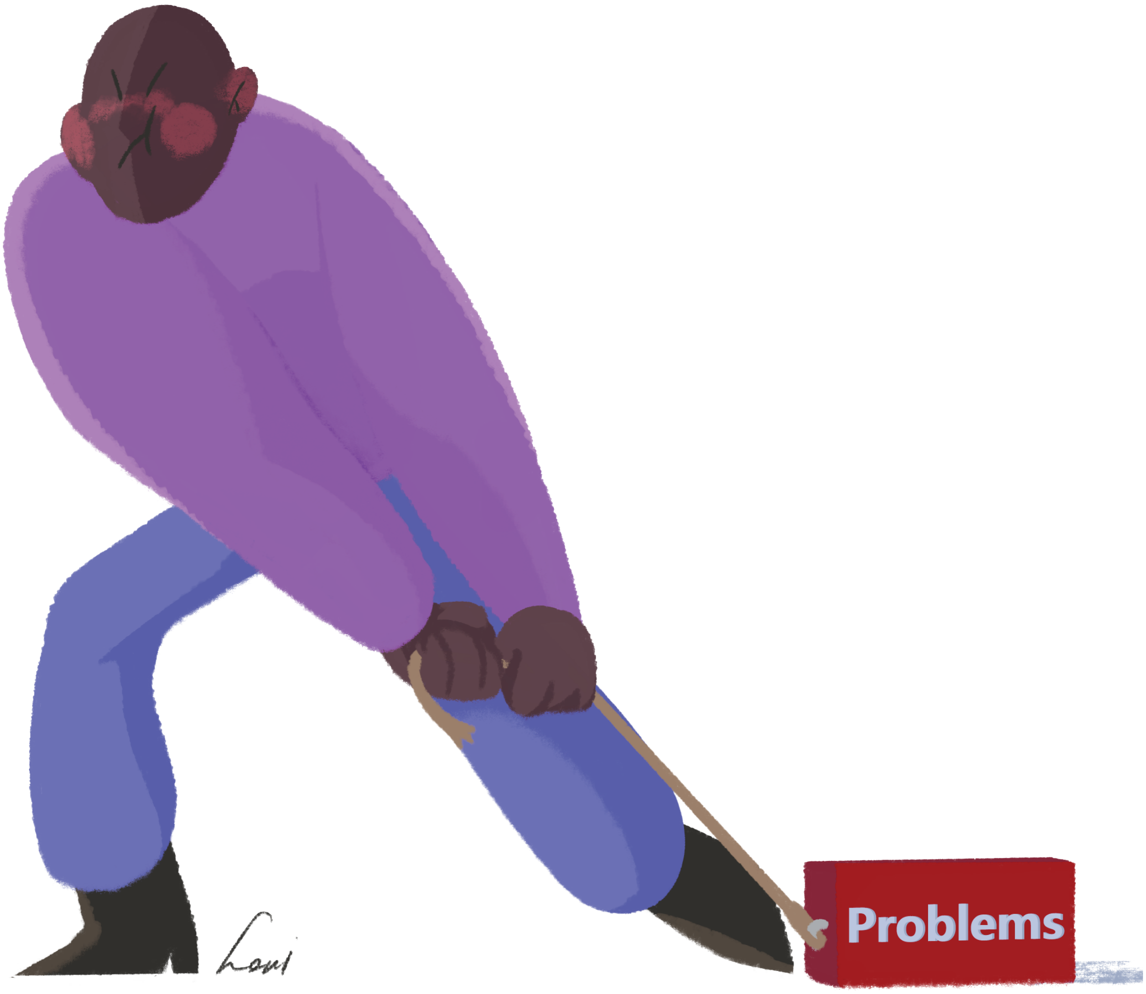

> Problems weigh you down.

Exercise 2b

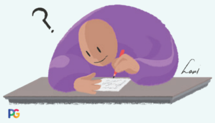

✓ Think back about the past week.
How much did you suffer from your mental health problems?
Draw a circle around the number.

1 Not at all **2 Hardly at all** **3 A bit** **4 A lot** **5 An awful lot**

✓ **How did you feel this past week?**
Draw a circle around the face.

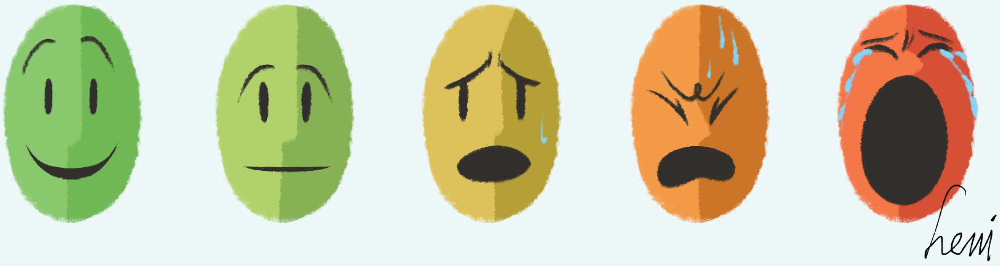

Mental health problems affect how you feel and what you do.

Exercise 2c

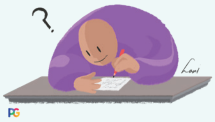

People cope with their problems in different ways.
Not all ways of coping help recovery.
Tick the box. What do you that not does help recovery?

- ☐ Drink alcohol
- ☐ Do drugs
- ☐ Withdraw
- ☐ Seeking a lot of contact
- ☐ Hurt yourself
- ☐ Hurt someone else
- ☐ Stop taking medication
- ☐ Take too much medication
- ☐ Smoke too much
- ☐ Eat too much
- ☐ Stop eating

- ☐ ....

- ☐ ....

Chapter 3 Strengths

When you have mental health problems, you sometimes forget
what you are still capable of. Beside your problems you also have strenghts.
You have mental health problems, but you also have strengths.
Strengths are what helps you forward, what you are good at or what you enjoy doing. They are about what goes well or works out for you.
For example, something you like doing or a good character trait that you have.

Exercise 3a

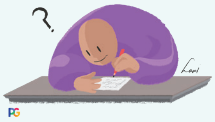

✓ **What are your strengths (tick the boxes)**

Qualities;

- ☐ I look after myself properly
- ☐ I am independent
- ☐ I am friendly
- ☐ I am hard-working
- ☐ I am grateful
- ☐ I don't give up easily
- ☐ I expect things to go well mostly
- ☐ I am good at listening
- ☐ I take other people's feelings into account
- ☐ I think properly about choices
- ☐ I am patient
- ☐ I have understanding for other people
- ☐ I respect other people
- ☐ I find it easy to talk to other people

- ☐ I am funny
- ☐ ☐ I stand up for myself
- ☐ ☐ I am calm
- ☐ ☐ I come up with my own ideas
- ☐ ☐ I am serious
- ☐ ☐ I stick to the rules
- ☐ ☐ I know how to enjoy myself
- ☐ ☐ I am honest
- ☐ ☐ I am brave
- ☐ ☐ I can handle criticism
- ☐ ☐ I am accurate
- ☐ ☐ I like learning new things
- ☐ ☐ I am curious
- ☐ ☐ I do things enthusiastically
- ☐ ☐ I think up new things
- ☐ ☐ I am not afraid to give my opinion
- ☐ ☐ I am spontaneous
- ☐ ☐ I am adventurous
- ☐ ☐ I am content
- ☐ ☐ I admit my mistakes
- ☐ ☐ I can forgive people
- ☐ ☐ I work well with my hands
- ☐ ☐ I am interested in other people
- ☐ ☐ I care for other people
- ☐ ☐ I am reliable
- ☐ ☐ I am self-confident
- ☐ ☐ I want to help other people
- ☐ ☐ I am sporty
- ☐ ☐ I am active

- ☐ I am honest
- ☐ I don’t give up easily
- ☐ I get on well with other people
- ☐ I don’t get angry easily
- ☐ I am creative
- ☐ I am good at working with other people

List your top 3 strengths:

1 ....

2 ....

3 ....

Exercise 3b

✓ Ask someone else what they think your good points are. (Phone someone or text them with this question.)

....

....

✓ Write down a compliment here that the person you are filling this in with gave you.

....

....

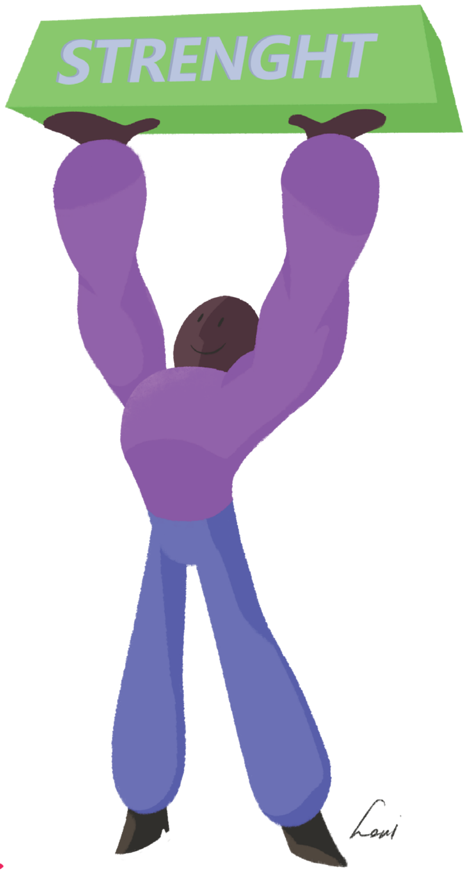

> Knowing what you’re good at.

Chapter 4 Recovery goes up and down

When you get treatment, you often hear the word 'recovery'.
Recovery doesn't mean you don't have any problems anymore.
That can happen of course, but most people still have some problems.
Recovery means learning how to deal with your mental health problems.
And learning what your strengths are.

Recovery can take a long time.
It is a process with ups and downs.
Your mental health problems might come back.
For example if you are under a lot of stress, are using drugs or stop your medication.
If your problems come back, you will know what to do to cope with them.

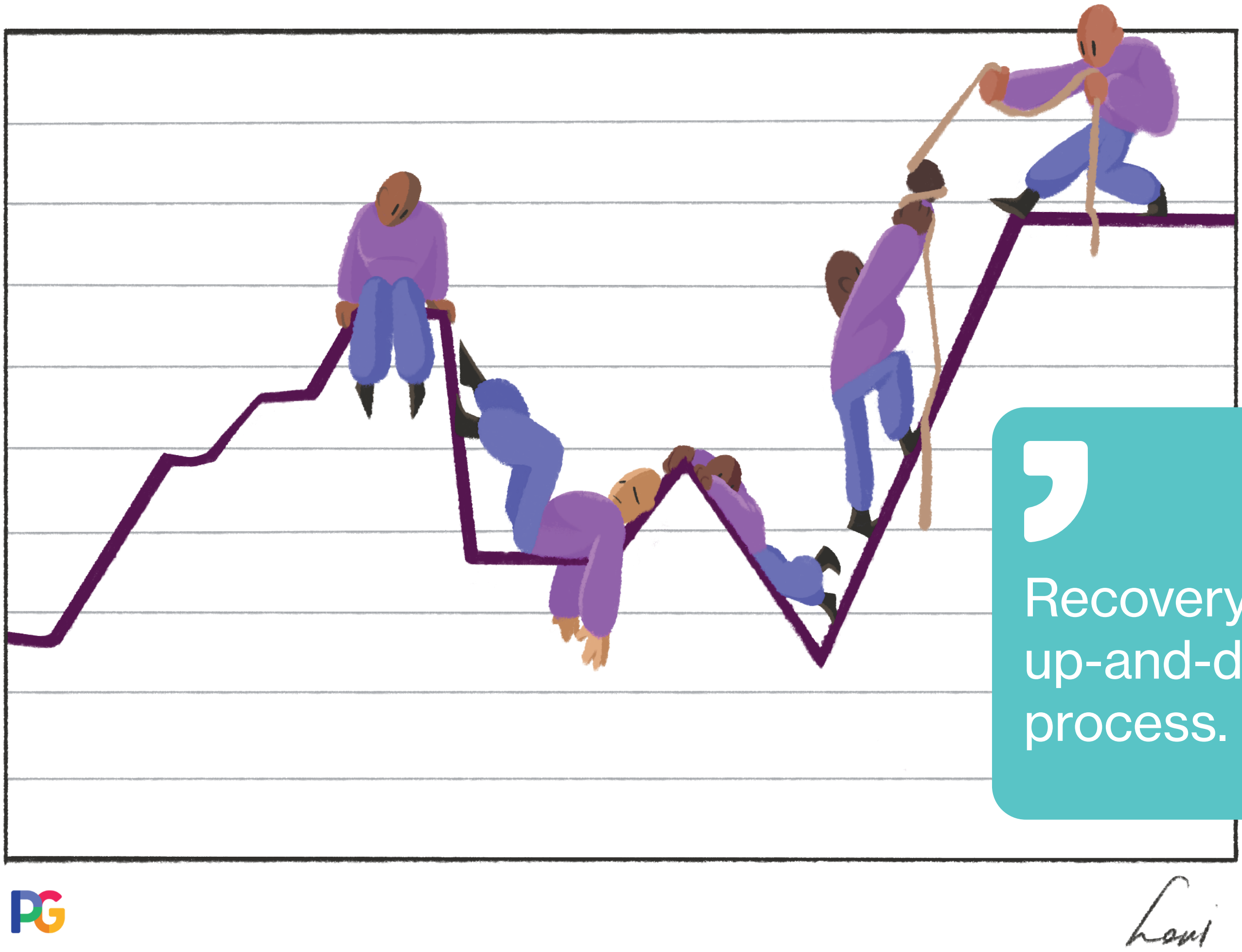

> Recovery is an up-and-down process.

Everyone has a different recovery process.
You will learn what helps and what doesn't.
You can influence your own recovery.

Exercise 4

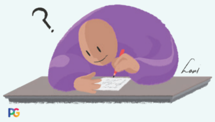

✓ **What can you do to make things go better?**

....

....

....

....

Chapter 5 Recovery takes time

You can't learn how to deal with your problems in one go.
Sometimes you make progress; sometimes you seem to be going backwards.
But you'll find you make more and more progress.

> Your problems are in charge.

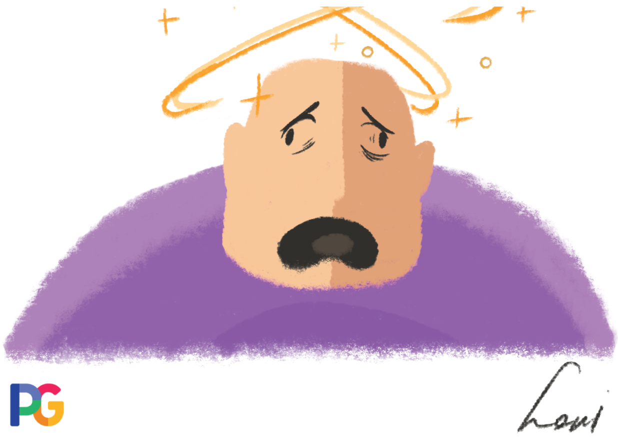

Step 1: Your problems are in charge

When you first get mental health problems, you don't understand what's happening.

You don't know what's happening to you.
It can make you scared, angry or sad.

You don't feel hopeful about the future.
You don't feel so confident.

Step 2: Fighting your problems

You don't want mental health problems.
You might feel ashamed.
You might feel guilty too.
You'd wish other people didn't know you have mental health problems.

> Fighting your problems.

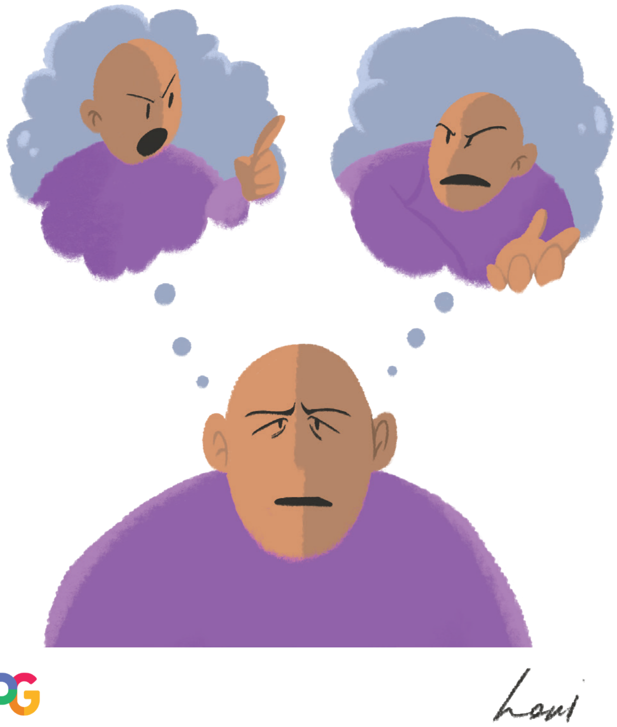

Step 3: Learning to live with your problems

You start to understand your problems.

You know you have mental health problems.

Or you know you tend to get mental health problems.

You become more confident.

You find ways of dealing with your problems.

You want your life back, even if you still have problems.

You start wishing things for the future.

> Learning to live with your problems.

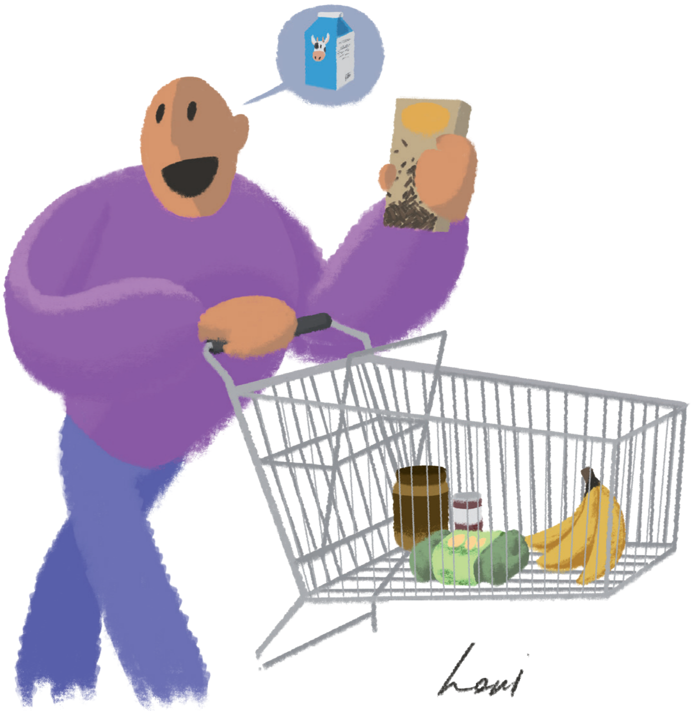

Step 4: Carrying on with your life

You feel like doing things.

You want to carry on with your life.

You have a daily rhythm.

You have things to do.

You know what to do if things get difficult.

You ask for help in good time.

> Carrying on with your life.

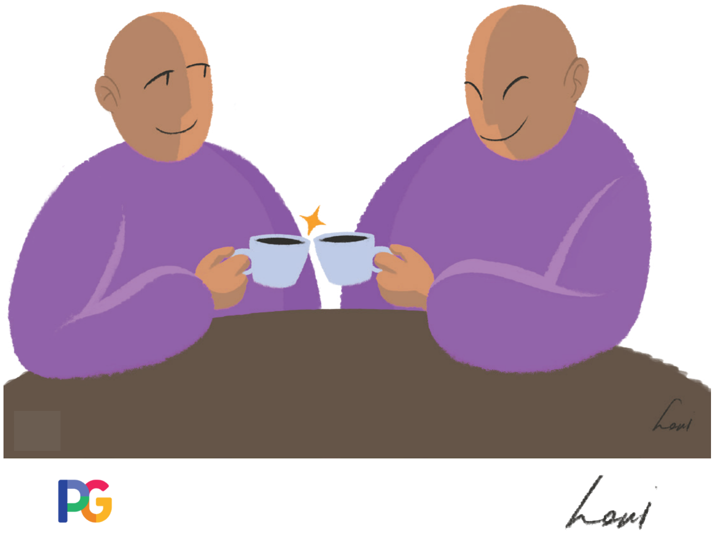

Exercise 5a

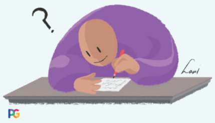

✓ Have another look at those steps.

Which ones do you recognize?

Which recovery step are you in?

Draw a circle round your answer

1 Your problems are in charge
2 Fighting your problems
3 Learning to live with your problems
4 Carrying on with your life

✓ **Why do you think you're in that step?**

....

....

....

Exercise 5b

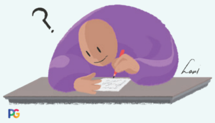

This helps me in my recovery.

Tick the boxes for the things you think are important.

- ☐ Sharing my experiences with other people (group therapy, people in the same situation as me)
- ☐ Having a friend
- ☐ People understanding what I've been through
- ☐ Hope for the future

- ☐ Accepting my problems
- ☐ Knowing what to do if my problems get worse
- ☐ Using medication
- ☐ Looking after myself properly
- ☐ Being in contact with my family
- ☐ Talking to my therapist
- ☐ Working
- ☐ Volunteering
- ☐ Organised daytime activities
- ☐ Having a hobby
- ☐ Sport or exercise
- ☐ Eating healthily
- ☐ Having pets
- ☐ Being outdoors in the countryside
- ☐ Having my finances in order
- ☐ Not getting into debt
- ☐ A nice relationship (partner)
- ☐ Being busy/engaged with my religion
- ☐ Getting compliments from other people
- ☐ Sleeping well
- ☐ Getting up on time
- ☐ Keeping to agreements
- ☐ Arriving on time

- ☐ ....

- ☐ ....

- ☐ ....

Chapter 6 Types of recovery

This is a longer section.
So take your time.

Recovery is about more than mental health problems.
It is also about carrying on with your life.
It is about making progress.
Sometimes you go back a bit.

We divide recovery into 4 types:

✓ **Finding out who you are**
✓ **Dealing with your problems**
✓ **Taking part again**
✓ **Getting involved again**

Finding out who you are

You are going to find out who you are again.
You will find out what you enjoy,
what makes you happy,
and what is important to you.
You will investigate how to do this.

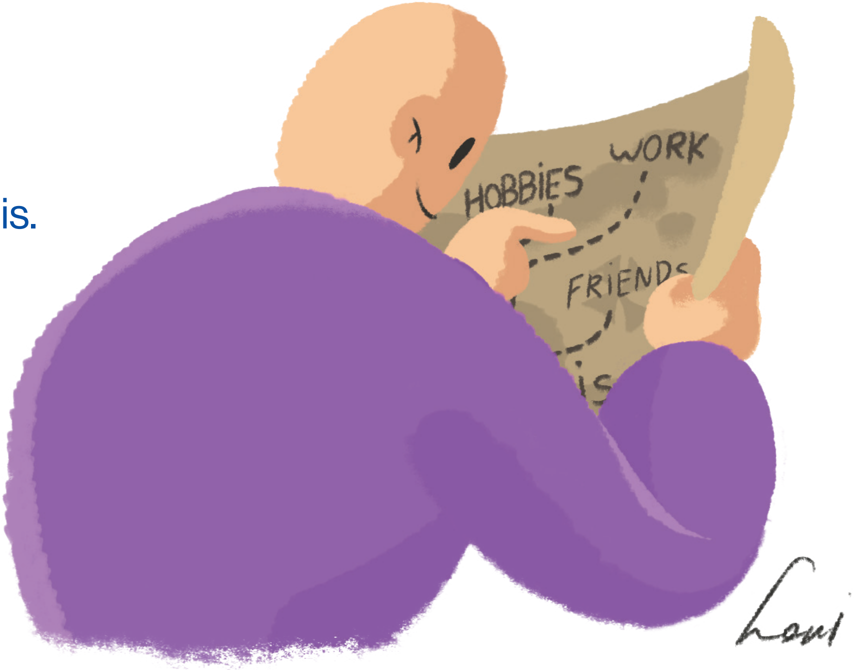

> Finding out what I want.

Exercise 6a

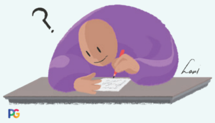

✓ Think of something you like.
What gets you out of bed in the morning?

☐ ....

☐ ....

☐ ....

☐ ....

☐ ....

✓ **What is important to you in your life?**
Try to name at least one thing (or more if you want).
Or name something you used to find important.

☐ ....

☐ ....

☐ ....

☐ ....

☐ ....

Dealing with your problems

You don't suffer as much from your problems.
You've learned how to deal with them.
You know what to do if you go through a difficult period.
Sometimes your problems will go away altogether.

Exercise 6b 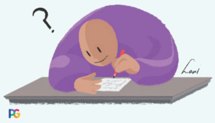

✓ Take a look at Exercise 2a on page 9.
Which problems do you want to go away?

....

....

✓ **Which problems are not so bad now as they used to be?**

....

....

✓ **What did you do to make your problems easier to cope with?**

....

....

Taking part again

You are going to pick up your daily life again.

For example, you are going to try to get into a regular day-night rhythm:

- ✓ Go to bed on time
- ✓ Get up on time
- ✓ Eat at fixed times
- ✓ Stick to agreements
- ✓ Do activities on fixed days.

You will also investigate what you can do on your own and where you need help.
Nobody can do everything on their own.
You will think about what help and support is best for you.
And who can help you.

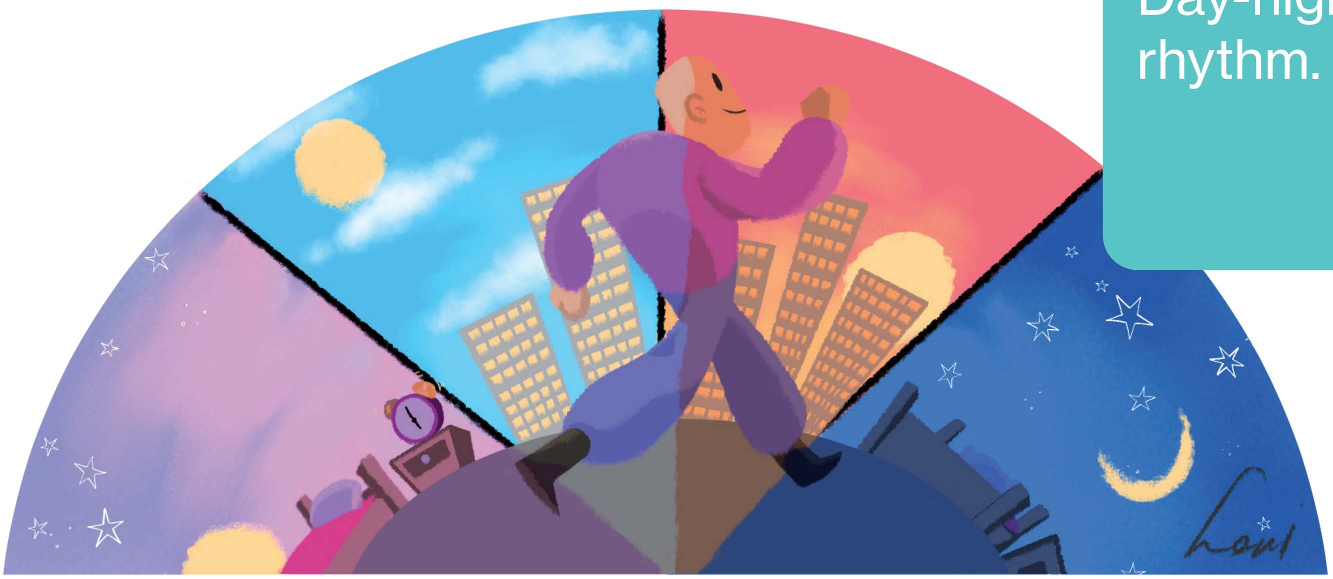

Day-night rhythm.

Exercise 6c

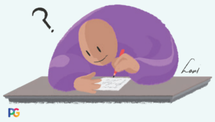

✓ Talk together about how things are going now.
Draw a circle around how things are going **now**.

I go to bed on time: Yes / No

Comment:...

I get up on time: Yes / No

Comment:...

I eat at fixed times: Yes / No

Comment:...

I have fixed appointments in the week: Yes / No

Comment:...

I stick to my agreements: Yes / No

Comment:...

I turn up to my appointments on time: Yes / No

Comment:...

Getting involved again

You are going to get involved in the community again.
There are many different ways of getting involved.

- ✓ School
- ✓ Voluntary work
- ✓ Sport
- ✓ Joining a club
- ✓ Work
- ✓ Organised daytime activities

You come into contact with other people.
You rediscover what you can do, what you are good at and what you enjoy.

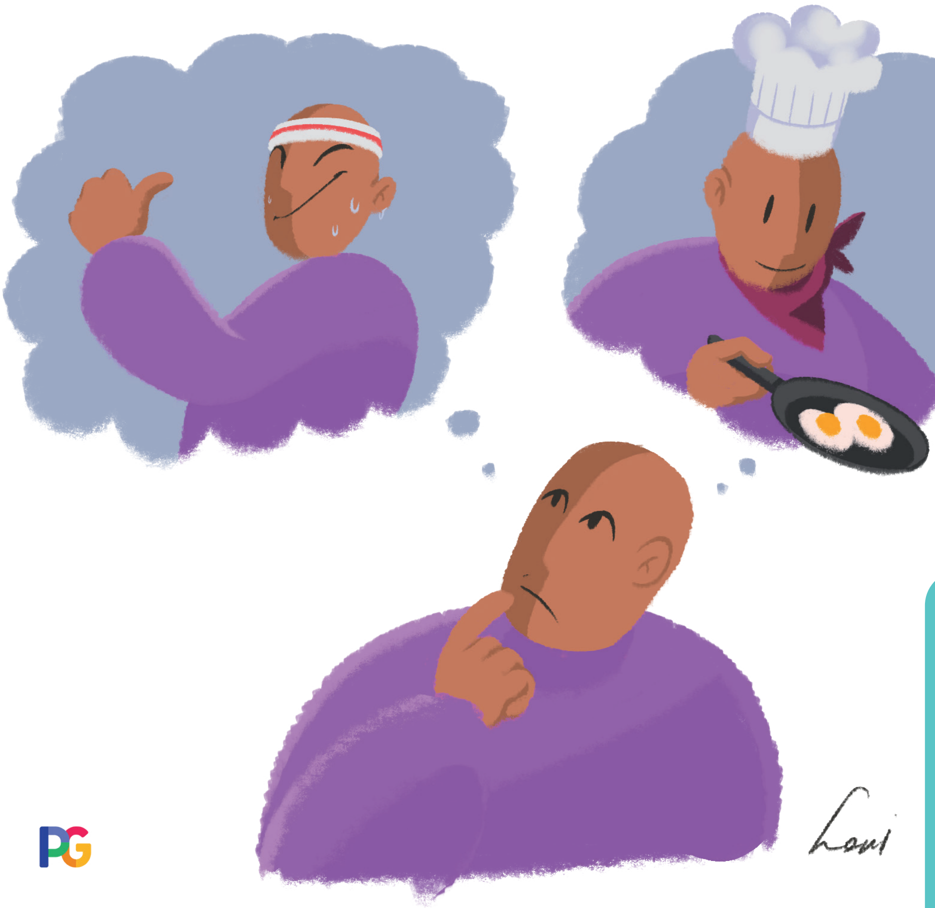

Thinking about activities.

Exercise 6d

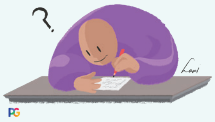

✓ Who do you have contact with?

Tick the boxes for your social contacts.

You can write down some notes too.

- ☐ mum/dad
- ☐ brother/sister
- ☐ son/daughter
- ☐ partner
- ☐ neighbour
- ☐ flatmate
- ☐ co-worker
- ☐ friend
- ☐ sports buddy

- ☐ ....
- ☐ ....
- ☐ ....
- ☐ ....
- ☐ ....
- ☐ ....

✓ What would you like to change about your social contacts?

....

....

....

....

✓ How can you do that?
(For example: reading bedtime stories to your son/daughter, do something fun with your sister, buying groceries for your neighbour.)

....

....

....

....

Chapter 7 Recovery plan

You already know what is going well.
You also know what you still want to work on.
It is important to do that step-by-step
in this way you work towards something.

For example:
a wish for the future.
a goal for the longer term.

Exercise 7a

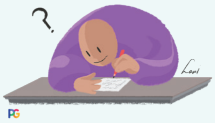

✓ **Write down what you want to change.**
What do you need to make that change?

1. I want:

....

....

To do this, I need:

....

....

2. I want:

....

....

To do this, I need:

....

....

3. I want:

....

....

To do this, I need:

....

....

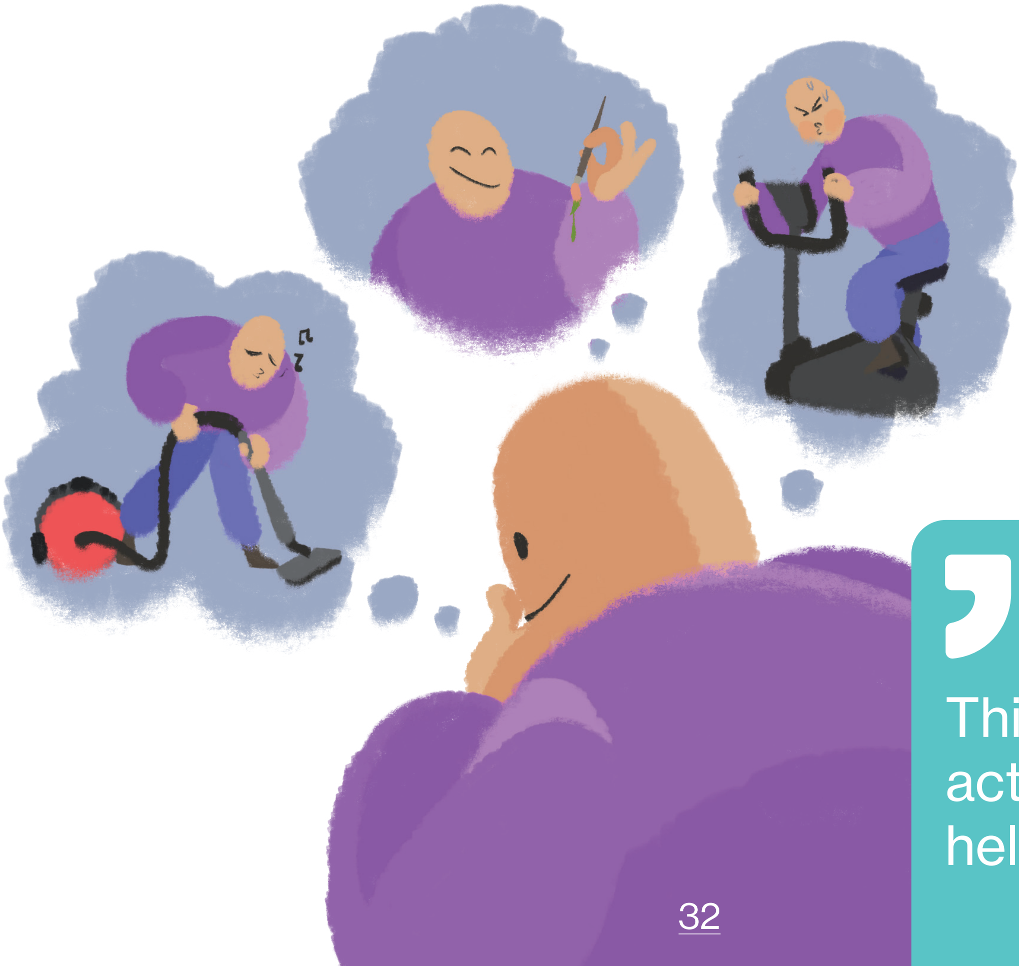

Think about activities that help.

Exercise 7b

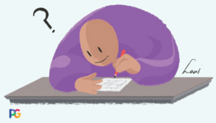

✓ Sometimes things don't go so well.
What can you do then to make things go better?
(Take a look at Exercise 5B on page 22.)

....

....

....

....

Exercise 7c 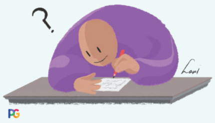

✓ **What can you do together with someone else to make things go better?**

....

....

....

....

....

Chapter 8 Coming to a close

You are nearly at the end of this booklet.
Have another chat about what you have learned.
The following exercise will help.

Exercise 8

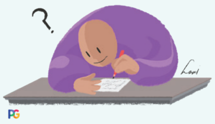

✓ What is the most important thing you learned from this booklet?

....

....

....

✓ What do you want to remember in future?

....

....

....

You have now come to the end of the booklet.
You have learned about your own recovery.
You may still have days when you feel bad,
or your problems might feel a bit worse.
If so, pick up this booklet and look at what you wrote down.

Have trust that things will get better again.
Believe in yourself.

Thanks for all your hard work.
You can share your recovery plan with the people who are important to you.
For example a relative, a friend or a mentor.

Because recovery is easiest when when you do it with other people.

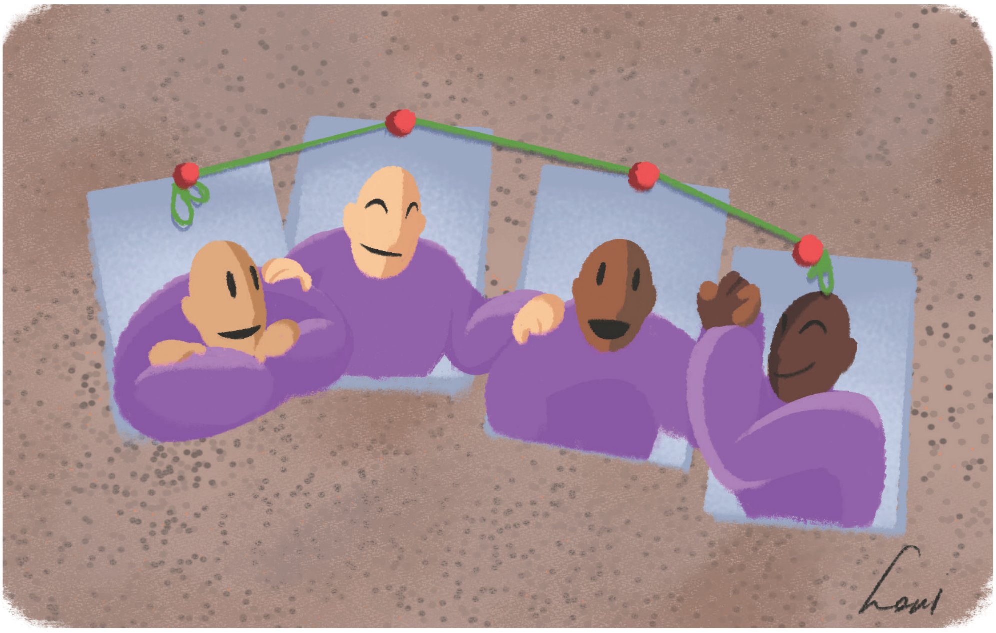

Recovery works better together.

Writing space for yourself

Writing space for yourself
