# Supplementary material for: Developing a recovery-oriented intervention for people with severe mental illness and an intellectual disability: design-oriented action research
Source: Front Psychiatry. 2023 Jul 19;14:1184798. doi: 10.3389/fpsyt.2023.1184798 (PMC10395094; doi:10.3389/fpsyt.2023.1184798)
Supplement: Supplementary file 2 [file Table_1.docx]

|  | Who? | What? | How? |
| --- | --- | --- | --- |
| Cycle 0 | Research team | Design first prototype | Based on literature |
| Cycle 1  Mental health professionals  (n=8) | Expert by experience 1  Social work 2  Nurses 4  Clinical psychologist 1 | Evaluate intervention, provide input for next prototype | One focus group |
|  | Research team | Revision intervention  New Prototype | Reflection  Log keeping and minutes |
| Cycle 2  Experts  (n=19) | Experts by experience:   - Mental health 2 - Family 2 - Intellectual disability 5   Psychiatrist 3  Developmental disability Physician 1  Orthopedist 1  Psychologist 2  Neuropsychologist 1  Nurse practitioner 1  Social Worker 1 | Evaluate intervention, provide input for next prototype and manual | Interviews |
|  | Research team | Revision intervention  New Prototype | Reflection  Log keeping and minutes |
| Cycle 3  Clients  (n=7) | Clients with severe mental health problems and mild intellectual disability (3) or borderline intellectual functioning (4) who already followed recovery focused treatment | First evaluation intervention by clients, provide input for next version | Two focus groups (n=4) and (n=3) |
|  | Research team | Revision intervention  New Prototype | Reflection  Log keeping and minutes |
| Cycle 4  Clients  (n=19)  Professionals (n=15) | Professionals FACT-teams Antes and their individual clients with severe mental health problems and mild intellectual disability (8) / borderline intellectual functioning (11)  Nurses 8  Social work 5  Psychologist 1  Expert by Experience 1 | Evaluation intervention, input for manual | Self-assessment forms |
|  | Research team | Revision intervention  Definitive Prototype | Reflection  Log keeping and minutes |

Supplementary Material 1. Table of cycles and participants
