# Supplementary material for: Developing a recovery-oriented intervention for people with severe mental illness and an intellectual disability: design-oriented action research
Source: Front Psychiatry. 2023 Jul 19;14:1184798. doi: 10.3389/fpsyt.2023.1184798 (PMC10395094; doi:10.3389/fpsyt.2023.1184798)
Supplement: Supplementary file 3 [file Table_2.docx]

|  |
| --- |
| Introduction   - Explanation definitions (mild intellectual disability or borderline intellectual functioning, severe mental health problems) - Explanation research - Reading break intervention   Key questions (four main themes)   1. Content  - Talking about recovery: which items are important and should be included? - Points of improvement  1. Assignments/exercises  - Which assignments fit within this intervention and are educational/fun/challenging/appealing? - Points of improvement  1. Understandable language  - Which language use is appropriate for this intervention? - Points of improvement  1. Visual support  - At what time does visual support add value and do the pictures support the text? - Points of improvement   Structure and lay-out   - Points of improvement   Closing questions   - Important topics not covered - Resume |

Supplementary Material 2. topic list used in focus groups and interviews with participants
