# Supplementary material for: Developing a recovery-oriented intervention for people with severe mental illness and an intellectual disability: design-oriented action research
Source: Front Psychiatry. 2023 Jul 19;14:1184798. doi: 10.3389/fpsyt.2023.1184798 (PMC10395094; doi:10.3389/fpsyt.2023.1184798)
Supplement: Supplementary file 4 [file Table_3.DOCX]

| Clients   - What did you think of the content of chapter 1,2,3…? - What did you think of the drawings? - How did you find the assignments? - Was the use of language clear?   Professionals   - How many sessions did you need to finish the intervention? - What did you think of the manual? - What did you like about this intervention? - What did you run into? - Do you have points for improvement? - What do you think this could bring in treatment. - Can you also extract things from this intervention that you can incorporate in the treatment plan and/or signalling plan? |
| --- |

Supplementary Material 3. Questions self-assessment forms for participants
