# Supplementary material for: Developing a recovery-oriented intervention for people with severe mental illness and an intellectual disability: design-oriented action research
Source: Front Psychiatry. 2023 Jul 19;14:1184798. doi: 10.3389/fpsyt.2023.1184798 (PMC10395094; doi:10.3389/fpsyt.2023.1184798)
Supplement: Supplementary file 5 [file Table_4.DOCX]

| Date | Content | New appointments | Present |
| --- | --- | --- | --- |
| 27-3-2020 | Adjustments to prototype after 10 interviews with experts | - Create an introduction. The word ''introduction'' becomes explanation. The word is clear and has simplicity; - Invitations sent to experts to review this new prototype again. First appointments scheduled. | Anne  Margot Ingeborg  Michiel |
| 15-5-2020 | Adjustments to worksheet after 15 interviews with experts, of which the last three were experts by experience intellectual disability | - Word recovery is a tricky word according to ID experience experts. Word recovery difficult to replace; - Word worksheet replaced with booklet. - Phases are given names for clarification. | Anne  Margot Ingeborg |
| 7-12-2020 | Online consultation with Lisette van der Meer | - Advisable to start with focus groups to gather general opinions and impressions of patients before presenting to individual clients; - Focus groups scheduled | Lisette  Anne  Margot Ingeborg |
| 11-04-2022 | Evaluation cycle 4 | - Falling puppet: Most participants recognize themselves in the drawings (see quotes). Unlike experts who thought that was too negative or abstract > voice client counts more > stays in; - Chapter 3 ‘’Strengths’’ is expanded because it is quite small compared to others while everyone seems to think it is an important chapter, both clients and professionals. - Add list of examples strengths. | Anne  Michiel  Ingeborg |

Supplementary Material 4. Example excerpts from the logbook
