# Supplementary material for: Developing a recovery-oriented intervention for people with severe mental illness and an intellectual disability: design-oriented action research
Source: Front Psychiatry. 2023 Jul 19;14:1184798. doi: 10.3389/fpsyt.2023.1184798 (PMC10395094; doi:10.3389/fpsyt.2023.1184798)
Supplement: Supplementary file 6 [file Table_5.DOCX]

| CYCLE 1  Mental Health care professionals | | |
| --- | --- | --- |
| Content | Participants mentioned to miss subjects: addiction, self-stigma, sexuality and assertive behaviour. Social roles should get more attention because it emphasizes roles beyond those of being a psychiatric patient. In addition, participants indicated that acceptance of vulnerability instead of full recovery is important to talk about. Recovery is not a word often used in this group of clients. One expert by experience mentioned that recovery is a personal process that takes years and cannot be achieved in a number of sessions.  Also several adjustments in the structure of the intervention were proposed, such as more images, less text and larger writing space. | |
| Exercises | The designed exercises required adjustments according to most participants. The true-or-false questions at the end of the intervention resembled a test. Participants suggested to add a scoring scale with emoticons/faces that could be used to rate the severity of psychological symptoms. | |
| Use of language | Participants additionally noted that the intervention entails many questions that are asked in succession. In general there was too much text, but the short sentences read well. | |
| Visual Support | All participants agreed that the number of pictures should be increased, but that this pictures should not be too crowded. Overestimation of the patient’s imagination should be prevented. Participants agreed upon the importance to add pictures of a person among other people as well as a picture about stress and vulnerability. | |
| **CYCLE 2**  **Experts** | | |
| Content | Three experts by experience ID missed an introduction of who you are.  *“Maybe a part where you write down what you like and tell a little bit about yourself and who you are.”*  The chapter “Introduce yourself” was therefore inserted at the beginning. Experts by experience who later assessed this chapter were positive about this.  In the section “examples of psychological complaints: tick what you recognize” the following items were missing: thinking about bad events from the past, thinking too much and being confused. The kind of coping people use was also missed.  Clumsy coping, such as using alcohol and drugs also had to be added.  Two experts made critical comments about talking to clients about the concept of recovery. They believe that we should not teach clients our concepts but that we should speak the language of the clients.  Almost all experts without an expert by experience background discussed about how to talk about “psychological complaints’’ or being “vulnerable to psychological complaints”, “be able to accept” or “that it’s just asking too much of people to be able to accept this complaints”. Experts by experience themselves were not concerned with the precise choice of words. They thought it was especially important that it was clearly and understandable. Two expert warned against too much text an nuance and advised to delete entire pieces of explanation.  Explanation about different roles you fulfil in life was too abstract. It has been replaced by an inventory of who you are in contact now and, what you would like to change. | |
| Exercises | For the question “how much trouble did you have with your psychological complaints last week” a 10-point scale was used. Requesting a week back was experienced as long by some experts, but other experts called it common in mental health settings.  One exercise consisted of filling in a doll with two sides: complaints and strengths. Most experts found this confusing and difficult. Complaints had already been mapped out in an earlier chapter and it was a double issue. The questioning of strengths was considered important but no doll was needed for that. The exercise “this helps with my recovery” gets no points for improvement. Even after adjustment, experts believe that the closing questions come across as a test. | |
| Use of language | Despite the attention that had already been paid to clear language, experts still had many points for improvement. Experts in the field of inclusive language indicated to start each sentence on a new line, not to use questions in the text, not to use comma’s, and, not to use connecting words that make a sentence longer. Experts also pointed to too much nuance and duplication.  *“We often add words that indicate nuance: very, a lot, shorter or longer time……that is because we often want to be too complete.”*  The four stages of recovery and the four types of recovery were confusing and misunderstood. It was considered important to use other words.     \| **Types of recovery** \| \| \| --- \| --- \| \| ***Regular use of language*** \| ***Easy language*** \| \| Personal recovery \| Looking for who you are \| \| Symptomatic recovery \| Dealing with complaints \| \| Recovery from daily life \| Rhythm and regularity \| \| Social recovery \| Join again \| \| **Stages of recovery** \| \| \| ***Regular use of language*** \| ***Easy language*** \| \| Stage 1 Being overwhelmed by the condition \| Step1 Your complaints are the boss \| \| Stage 2 Struggling with the condition \| Step 2 Struggle with your complaints \| \| Stage 3 Living with the condition \| Step 3 Learn to live with your complaints \| \| Stage 4 Live beyond the disease \| Step 4 Move on with your life \| \|  \| \| | |
| Visual Support | In general experts liked the pictures but some were very complicated. Especially some pictures with the head loose on the torso were judged as too abstract, unhelpful or even violent. The head just had to be on the torso and the explanation behind it was experienced as too abstract and far-fetched.  Also, some pictures were too metaphorical, so there was a risk that they would be insufficiently understood. For example, this came back to the doll with weights in his hands, depicting being in balance. Another example was the picture where the doll could choose between different caps to portray different roles in life.  Experts by experience ID looked very critically at facial expressions. Frequently heard criticism was that the doll looked angry or not happy.  One expert thought the pictures were a bit masculine. Several experts expressed themselves positively about the use of different skin colours. The pictures were not experienced as childish.  One expert explained the principle of using pictures. A picture can be didactic or brightening, but in the latter case may not be disruptive in the line of the text. Another principle is that the pictures are accepted by those who are addressed, in this study, the clients. | |
| **CYCLE 3**  **Clients with SMI and MBID** | | |
| Content | As examples of inadequate coping we mentioned drinking alcohol, using drugs, isolating yourself. All clients missed other subjects such as take too much medication, do not use medication, too much smoking, eating more and, stop eating and advised to add this. The item about the different social roles a person could have in his life was not understood by almost all clients. None of the clients were able to summarize the different types of recovery after reading this chapter because the chapter has no clear structure. In general, all participants had a lot of comments to the structure of the intervention and the lay-out.  *“What I also like is to work with those dots. […] What am I good at? You only have one line, but who says that’s enough for me?”* | |
| Exercises | All clients were positive about the introduction assignment ‘’ Tell who you are, what you like and what you are good at’’. They described the assignment as fun. One client feels that there are too many options when it comes to the question about psychiatric complaints. Others find it a clear assignment. They all understand the faces scale well.  Mentioning own strengths is difficult for all clients. They didn’t know how to approach the assignment, even after explanation. It would help to mention nice things and activities from the past and to talk about it, “maybe you’ll find out then”.  The checkbox to tick what helps with recovery is clear and considered as a good assignment. Clients also think it is important for professionals to know this, because then they can better support them.  Other assignments are also experienced as useful and clear, for example the assignment about the different stages of recovery, the yes/no questions in the paragraph rhythm and regularity, and an open question as ‘’What do you like to get out of bed for?’’ Some clients were able to formulate goals for their recovery plan while others felt stable and wished to maintain this stability. | |
| Use of language | Clients had no problems with the choice of words psychological complaints and they wonder what else to call it. One client explained it as “have no meaning in your life anymore, loneliness and things like that”. One other client felt the sentence “you also have psychological complaints” sounded hard, but she thought it was the truth at the same time. All clients agreed with her.  The word clumsily in the sentence ”sometimes you handle complaints clumsily, for example (multichoice) drinking alcohol, using drugs, …etcetera” is not well understood by some clients. The think it sounds weird, like you’re dropping something.  One client describes the text in the chapter looking for who you are as “a thinking text” and “a C-level text instead of A-level”: you will re-examine how to give important things a place in your life again.  Searching for a clearer explanation for social roles that you can fulfil in your life (be a father, a neighbour or an employee), all clients agreed that the concept of having social contacts would be easier to understand.  They unanimously shared the opinion that the text is generally clear and easy to understand. | |
| Visual Support | In general, clients sometimes found the pictures very clear, others were not understood, and with still others they had comments and areas for improvement. A recurring theme was the puppet's facial expression, where the criticism was often that the puppet looks difficult, doesn't seem to feel like it, or doesn't look happy.  An example of a picture that is immediately understood is the picture of day and night rhythm. All clients reported that it was a beautiful picture. Also the picture (Picture 1.) at “your complaints are the boss” is directly understood:  *“As if he is a bit confused” and “that he has to take in too many things, because he has too many things in his head.”*  They think that picture fits the text well. This is not the case with the picture made in the section staying balanced. It shows the puppet with a pair of scales in its hands. Only a small number of clients understood the picture well, but others saw a bodybuilder in it and it did not support the text.  In the chapter ”moving on with your life” the corresponding picture was unclear. It depicted the puppet with a child in its arms, but the link with the text was made by a few.  In all cases the clients reported that the VAS (faces-scale) was clear and that they could put a circle around the most fitting face. The different mouths, some a line and others round, were distracting. | |
| **CYCLE 4**  **PILOT Individual mental health professionals and their clients** | | |
| **Clients** | | ***Professionals*** |
| Content | Clients valued talking about recovery and appreciated the attention paid to both complaints and strengths. One participant said she recognized a lot of the complaints. The chapter on strengths was found difficult and troublesome, but also interesting. All participants found it nice and important to talk about. Nobody had negative feedback about the content:  *“Tough to think about things I'm good at. That's unknown to me. It's a good thing to do, though. In a lesser period you can read this back.”*  In the chapter ”recovery takes time” it was found interesting to know what recovery steps there are and in which step you yourself are.  Clients found it recognizable but also confronting. A number of participants mentioned that they had to read a lot in this chapter.  The types of recovery section is good in content, but is perceived by many participants as large and cluttered.  Clients were glad that there was attention for steps they can take in the future. One client went into resistance, furthermore clients expressed that they had learned a lot or have gained insight in what they had already achieved. Some clients also said something about the structure of the chapters, namely that it became more difficult, more personal, and also more relevant as the chapters progressed. | Professionals sometimes had to give examples of strengths, but they found it a very positive approach, appropriate for positive psychiatry.  Professionals agreed that the steps of recovery are helpful, but sometimes confrontational, but it helped professionals to give substance to the conversation and helped the client to gain more insight into themselves.  Professionals agreed that the content was clear and important and could lay the foundation for a recovery plan or signalling plan.  Professionals expressed positive views on the topics covered:  *“Themes that are self-evident do not normally always come up in a treatment. By following this module they do.”*  They did run into the fact that one chapter was larger than another, that clients did need direction for varying reasons (e.g., answering open-ended questions), or that chapter and assignments had the same type of answer (knocking on an open door).  Professionals gave several points for improvement such as a double sentence, page layout and suggested a digital version. All professionals felt that the method contributes to increasing the understanding of the client and the professional and that the outcomes can be adopted in the treatment plan and crisis plan. |
| Exercises | The first assignment was an introduction assignment: tell who you are, what you like and what you are good at. One person thought it was childish, many people thought it was fun, nice to introduce themselves but five people found it hard to name what they were good at.  The second consideration, ticking off complaints, was clear but also quite confronting. Because many of the complaints were recognizable, many participants found it pleasant to be able to talk about them. Mentioning one's own strengths was difficult for all participants, but also nice to think about. One client, when asked the open-ended question ''What can you do to make things better?'' indicated that she needed examples, others succeeded. In the fifth chapter, clients had to indicate their stage of recovery:  *“Exciting to see that I am in step 3 of recovery”*  and then tick what helps them in their recovery. Most clients found the list helpful and nice to fill out, but also difficult and confronting; a few found it too long. The recovery chapter types had four assignments to portray the current personal situation. Opinions differ on these assignments. Two clients mentioned that they saw that they were already doing many helpful things for their recovery. The assignments are “thinking assignments” and therefore sometimes a bit more difficult for some, and “fun and easy” for others. Three clients found it too many assignments. In Chapter 7, clients experienced thinking about what they want to change as pleasant and insightful.  In the closing chapter, repetition is perceived as helpful by clients and, they could clearly tell what they had learned. One client was completely fed up.  *“Fine to draw the conclusion that I can do it on my own. I just want to stop using weed.”.* | Chapter recover plan: professionals found this a ''forward-looking task.'' |
| Use of language | Some words had to be explained, especially to clients with Dutch as a second language. One client thought ‘’psychological complaints’’ was difficult.  Most people understand the language well:  *“I think the use of language is good. I understand what it says and what is expected of me.”*  In the assignment “Write down what you want to change. What do you need to do this?” two clients did not find it clear what to write down in the question, what do you need. The question was not understood. | Professionals automatically explained the words that clients did not understand and then checked if it was clear after this explanation. |
| Visual Support | The majority of participants founnd the drawings fun, helpful and clear except for minor areas of improvement. However, drawings didn't have to be as much for three clients or clients who indicated they weren't really paying attention. One person called all the drawings boring because there are no rainbow colors in them. Some persons found it annoying that it is only a male puppet. Clients looked very carefully at the facial expressions and often had criticisms and points for improvement here. Also, drawings were not always understood by everyone.  Surprisingly, a falling figure at the complaints section is much appreciated by some clients, whereas experts thought this would be too negative or abstract:  *“I feel like I am in the drawing.”*  A drawing was missed in the section on strength. Clients unanimously responded positively to the VAS (faces-scale). | Some drawings were found unclear, while others were very helpful:  *“’Helpful for clients to be able to visually see what stage of recovery they are in. Sometimes you are further along than you think.” (Professional)* |

Supplementary Material 5. Details of the results.
